# Role of computational tools and cultural values on academic engagement among university students in collaborative learning environments in China

**DOI:** 10.3389/fpsyg.2026.1885883

**Published:** 2026-08-24

**Authors:** Mingguang Duan, Xiaorui Lan, Yan Yang

**Affiliations:** 1 Innovation and Entrepreneurship Institute, Guangxi Normal University, Guilin, Guangxi, China; 2 School of Foreign Languages, Guilin College, Guilin, Guangxi, China; 3 Department of Chinese, Lyuliang University, Lyuliang, Shanxi, China

**Keywords:** academic engagement in higher education, computational tools in collaborative learning culture, cultural value and leadership skill, interdisciplinary problem-solving attitude, unique contribution

## Abstract

**Introduction:**

Technology-supported collaborative learning environments require higher education institutions to foster students' analytical, interdisciplinary, and collaborative capabilities. Although computational tools may support analytical thinking and problem-solving, their academic value may depend on students' learning orientations and interdisciplinary problem-solving attitudes. Similarly, cultural values may shape students' participation in collaborative activities through leadership skills and individual contributions. Accordingly, this study examines two complementary pathways associated with academic engagement: the pathway from computational tools to academic engagement through interdisciplinary problem-solving attitudes, and the pathway from cultural values to academic engagement through leadership skills and unique contributions.

**Methods:**

A cross-sectional survey was conducted among 502 university students in China. The proposed framework integrated technological, socio-cognitive, and collaborative dimensions of digitally supported learning. Seven hypotheses were empirically examined using primary survey data. Robust regression with heteroskedasticity-consistent standard errors was employed to account for potential heteroskedasticity, while bootstrap analysis was used to assess indirect associations under non-normal data conditions. The analysis focused on the associations among computational tools, interdisciplinary problem-solving attitudes, cultural values, leadership skills, unique contributions, and academic engagement.

**Results:**

The findings indicate that the effectiveness of computational tools was more strongly associated with students' learning orientations and interdisciplinary problem-solving attitudes than with technological exposure alone. In addition, the positive association between cultural values and academic engagement was statistically explained by leadership skills. Leadership skills showed a stronger positive association with academic engagement than unique contributions, emphasizing the importance of role-based coordination and collaborative leadership in digitally supported learning environments.

**Discussion:**

The findings suggest that academic engagement in technology-supported collaborative learning environments is associated with the interaction of cognitive readiness and socio-collaborative participation. Computational tools appear to be more meaningful when embedded within learning processes that strengthen interdisciplinary problem-solving attitudes, while culturally grounded participation may be associated with engagement through students' leadership capabilities. These findings highlight the importance of integrating technological competencies with socio-collaborative capabilities when designing higher education learning environments. Given the cross-sectional design, the reported relationships should be interpreted as statistical associations rather than causal effects.

## 1 Introduction

Over the past decade, higher education has increasingly embraced technology-supported pedagogical approaches that promote active collaborative learning through student-centered instruction. Computational tools (CT), including simulation software, coding platforms, collaborative applications, and data analytics systems, have become integral components of contemporary teaching-learning processes. Through the lens of Social Constructivist Learning Theory, recent technologies provide opportunities for problem solving through collaborative interaction, thereby supporting meaningful knowledge construction among learners (Vygotsky, 1978). Existing articles consistently report that the effective use of CT is positively associated with students' analytical thinking and their problem-solving ability (Kim and Kwon, 2024).

At the same time, collaborative learning turns an important instructional approach that suggests university students to pursue shared academic goals through peer interaction and collective responsibility (Chaudhry et al., 2024). Those environments are associated with higher-order learning outcomes, including critical thinking in leadership and academic engagement (AE) for university students. Nevertheless, there are very few empirical articles that examined the psychological processes behind CT-induced collaborative learning and AE, thereby creating a critical knowledge gap in this regard.

The pedagogical value of CT extends beyond their technological functionality. Successful integration of technology depends on meaningful pedagogical implementation and supportive learning environments (Montag et al., 2024). When appropriately placed in collaborative learning activities, CT are associated with interactive feedback and communication in a collaborative manner. Thus, students are able to participate more actively in shared learning experiences. Recent articles on educational psychology focus more on cognitive and collaborative processes, through which students acquire knowledge in digital world (Karakose et al., 2021).

In collaborative learning, interdisciplinary problem-solving attitudes (IPSA) represent an important socio-cognitive orientation. IPSA describes students' willingness to integrate knowledge across disciplinary boundaries and collaboratively develop solutions to complex academic problems (Yan et al., 2024). Social constructivist and collaborative learning theories discuss that CT-supported learning suggests students to build understanding through inquiry and collaboration (Laal and Ghodsi, 2012; Johnson and Johnson, 2009). Accordingly, CT are associated with stronger IPSA by facilitating joint analysis and collaborative exploration of problems (Lattuca et al., 2013). Even while contemporary literature separately demonstrates the educational benefits of CT amid collaborative learning, the time is ripe to examine whether IPSA is the psychological mechanism connecting CT-supported learning to students' AE.

Alongside technological factors, researchers recognize that university students' socio-cultural orientations and their cultural values (CV) affect learning experiences, particularly in culturally diverse educational institutions. Students enter higher education with different social norms and communication styles, which affect the interpersonal interaction (Ndasauka and Ndasauka, 2024). For example, university students from collectivist cultures often emphasize cooperation and group harmony, whereas pupils from more individualistic cultures place greater importance on autonomy and independent thinking. This diversity enriches collaborative learning, and sometimes leads to coordination challenges requiring adequate interpersonal communication (Dumitru and Chirleşan, 2025).

Accordingly, culturally diverse learning environments suggest intercultural competencies, such as empathy and adaptability (Thinh, 2025). Existing literature shows that CV are closely related to students' leadership behaviors and individual participation in collaborative learning. Therefore, there are ample scopes to investigate whether leadership skills (LS) and unique contributions (UC) represent theoretically proposed psychological tools linking CV with AE, particularly under CT-induced collaborative learning (Lomellini et al., 2025).

Taken together, students' AE may be affected by complementary cognitive and socio-cultural processes that work under CT-induced collaborative learning. Universities increasingly use blended and online learning environments, where digital tools support both live and delayed student communication (Pavo and Ángel, 2021). Researchers find that digital learning platforms support collaboration and interdisciplinary learning (Jiang et al., 2024), even while only very few articles combined cognitive and socio-cultural perspectives to explain students' AE in higher education. Accordingly, the present study addresses the following research questions:

- How are CT associated with IPSA and AE among university students in technology-supported collaborative learning environments, and to what extent do IPSA statistically account for the observed association between CT and AE?
- How are CV associated with LS, UC, and AE among university students in technology-supported collaborative learning environments, and to what extent do LS and UC statistically account for the observed association between CV and AE?

To address, the present study facilitates a theory-supported model that examines the associations among current constructs, namely CT, CV, IPSA, LS, UC, and AE, under CT-induced collaborative learning. Specifically, this study investigates whether IPSA are statistically associated with relationship between CT and AE, and whether LS and UC indirectly influence the relationship between CV and AE. Consistent with the cross-sectional approach, this study combines technological, cognitive, and socio-cultural perspectives to explore the psychological processes associated with university students' AE.

In rest of this paper, Section 2 provides a brief review of established theories along with recent literature on current constructs, and thereby frames the hypotheses of this research. Section 3 illustrates the research methodology to be adopted in current research, whereas Section 4 reports the results of various statistical tests on current research data. Furthermore, this examines the validity of seven proposed hypotheses. Then, Section 5 extracts a number of implementable acumen for stakeholders of this study. Furthermore, this identifies the limitations along with associated future scopes of research. Section 6 frames the conclusions of present research.

## 2 Conceptual framework and research hypotheses

This section provides a brief review of established literature associated with the present research, and thereby develops the proposed hypotheses successively, as below.

### 2.1 Computational tools and interdisciplinary problem-solving attitudes

The increasing integration of CT has transformed collaborative learning in higher education, as university students are found to engage in inquiry-based and interdisciplinary learning activities. From the perspective of Social Constructivist Learning Theory, knowledge is actively constructed through social interaction, dialogue, and collaborative meaning-making rather than passive transmission of information (Vygotsky, 1978; Powell and Kalina, 2009). CT is a mechanism that facilitates peer interaction and collective knowledge construction within authentic learning environments. The Problem-based Learning Theory argues that students develop higher-order cognitive abilities, when they collaboratively solve authentic and complex multi-disciplinary problems (Hmelo-Silver, 2004; Savery, 2015). Within these theoretical perspectives, CT extend beyond their technological functionality and become pedagogical resources that support collaborative inquiry, interdisciplinary reasoning, and knowledge integration across disciplinary boundaries (Nguyen et al., 2024a).

Contemporary educational research consistently demonstrates that technology-supported learning environments promote computational thinking and interdisciplinary collaboration. Also, CT-supported interdisciplinary collaboration guides university students to analyze complex problems more effectively by integrating knowledge from multiple disciplinary perspectives. In this regard, Zhou et al. (2021) reported that interdisciplinary collaboration within healthcare engineering enhanced participants' AE in analyzing complex real-world problems while strengthening collaborative decision-making. Moreover, studies conducted in computer science and STEM education have demonstrated that technology-supported problem-based learning significantly improves students' analytical reasoning and interdisciplinary learning attitudes (Kwon et al., 2021; Hava and Koyunlu Unlu, 2021). Thus, CT facilitate active participation in collaborative learning rather than merely supporting content delivery.

Recent advances in educational technologies have strengthened the educational role of CT. These modern systems facilitate students to explore authentic interdisciplinary problems collaboratively while simultaneously developing computational thinking and reflective reasoning (Longo et al., 2024; Kim and Kwon, 2024; Montag et al., 2024). Alkış Küçükaydın et al. (2024) identified twenty-first-century skills as an important explanatory mechanism linking computational thinking with positive STEM attitudes. In this area, Sun and Liu (2026) demonstrated that CT positively predicted students' attitudes toward generative artificial intelligence across different educational levels. They found that technology-supported cognitive development extended beyond disciplinary knowledge to broader learning orientations.

The pedagogical value of CT therefore lies in developing collaborative knowledge construction. Through interactive communication and exposure to multiple disciplinary perspectives, university students become more willing to integrate diverse forms of knowledge when addressing authentic academic problems. Those learning environments encourage university students to evaluate alternative viewpoints and construct comprehensive solutions, both of which are fundamental characteristics of IPSA. Accordingly, Social Constructivist Learning Theory (Vygotsky, 1978; Powell and Kalina, 2009) and Problem-based Learning Theory (Hmelo-Silver, 2004; Savery, 2015) jointly describe that CT-supported collaborative learning strengthen university students' willingness to improve IPSA.

Despite substantial evidence linking CT with IPSA, computational thinking, higher-order cognitive development, and interdisciplinary education, relatively limited empirical studies have explicitly examined IPSA as a distinct socio-cognitive outcome of technology-supported collaborative learning. Existing research has primarily focused on academic performance or collaborative learning outcomes, while only very few empirical studies examine whether CT are directly associated with university students' IPSA. Accordingly, this study proposes the following hypothesis:

*H*_1_: Computational tools are positively associated with interdisciplinary problem-solving attitudes among university students in technology-supported collaborative learning environments.

### 2.2 Computational tools and academic engagement

The AE is widely recognized as a multi-dimensional psychological construct reflecting students' behavioral participation and cognitive investment in learning activities (Fredricks et al., 2004; Kahu, 2013; Nguyen et al., 2024b). Established educational psychology literature considers AE a central determinant of learning quality, because highly engaged students demonstrate greater persistence with stronger self-regulation. Moreover, they can achieve deeper cognitive processing and superior academic achievement than their less engaged counterparts (Bond et al., 2020; Tu et al., 2023). Thus, rather than representing mere classroom participation, AE reflects students' active interaction with learning tasks and peers within the wider learning environment.

Theoretical relationship between CT and AE is primarily grounded in Student Engagement Theory (Fredricks et al., 2004), Social Constructivist Learning Theory (Vygotsky, 1978; Powell and Kalina, 2009), and Problem-based Learning Theory (Hmelo-Silver, 2004; Savery, 2015). Student Engagement Theory argues that meaningful learning occurs when educational environments promote academic participation through autonomous feedback-based authentic learning experiences (Kahu, 2013; Fredricks et al., 2004). Likewise, Social Constructivist Learning Theory proposes that knowledge develops through collaborative interaction and shared meaning-making, whereas the Problem-based Learning Theory emphasizes that authentic technology-supported problem solving motivates learners to become active participants rather than passive recipients of knowledge. Therefore, these theoretical perspectives suggest that CT create learner-centered environments where collaboration, inquiry, and reflective learning naturally develop higher levels of AE (Jiang et al., 2012).

Rapid expansion of CT has fundamentally transformed higher education by providing university students with interactive learning environments that facilitate collaboration and self-directed learning. Rather than functioning merely as instructional technologies, CT support learner-centered pedagogical practices that allow students to collaboratively construct knowledge through discussion. Those students make use of simulation software, intelligent tutoring systems, collaborative applications, learning management systems, and other tools (Nguyen et al., 2024a). In turn, these technology-supported environments encourage active participation and collaborative inquiry in learning activities, thereby strengthening students' behavioral and cognitive engagement.

Empirical evidence consistently demonstrates the positive contribution of CT to AE. During emergency online education, Abou-Khalil et al. (2021) reported that appropriately designed technology-supported instructional strategies significantly enhanced students' participation and learning experiences despite resource limitations. Alam (2022) found that intelligent tutoring systems, socially interactive robots, and artificial intelligence-assisted instructional technologies increased learner interaction and classroom engagement. Recently, Akpen et al. (2024) observed that interactive digital learning resources substantially improved students' AE even when improvements in academic performance remained comparatively modest. Thus, CT primarily influence learning processes before affecting achievement outcomes. Earlier systematic reviews likewise concluded that CT-based technologies generally exert positive effects on behavioral and cognitive engagement (Bond et al., 2020).

Recent technological advances have expanded the educational capabilities of CT, because this creates more engaging learning environments for university students (Kim, 2024; Zhang et al., 2023). Yan et al. (2024) associated generative artificial intelligence with improvements in meta-cognitive regulation and collaborative knowledge construction. Also, studies investigating chatbot-assisted learning reported that artificial intelligence-supported educational systems positively influence students' motivation, participation, self-regulated learning, and AE across higher education contexts (Rui and Jin, 2026). In a novel research, Chaudhry et al. (2024) demonstrated AE as an important psychological mechanism linking supportive learning environments with students' overall wellbeing.

Researchers describe that educational benefits of CT extend beyond facilitating interaction. University students can visualize complex problems, as they exchange ideas continuously with peers. They can also integrate multi-disciplinary knowledge and assume greater responsibility for their own learning. These characteristics promote autonomy and sustained participation, each of which is consistent with Student Engagement Theory (Fredricks et al., 2004) and Social Constructivist Learning Theory (Vygotsky, 1978; Powell and Kalina, 2009). Recent systematic reviews conclude that CT is positively associated with learning when this supports active learning, thereby reinforcing the importance of pedagogically meaningful technology integration (Nguyen et al., 2024b).

Despite growing empirical evidences linking CT with online learning, computational thinking, collaborative learning, and technology-enhanced pedagogy, there are ample scopes to investigate the relationship between CT and AE within technology-supported collaborative learning environments while simultaneously considering the cognitive mechanisms through which this relationship often operates. Unlike most existing articles considering AE as an educational outcome without integrating IPSA into the same theoretical framework, the present study proposes the following hypotheses:

*H*_2_: Computational tools are positively associated with academic engagement among university students in technology-supported collaborative learning environments.

### 2.3 Interdisciplinary problem-solving attitudes as the explanatory pathway

Building upon the relationships proposed in the preceding hypotheses, the educational benefits of CT are unlikely to translate into AE automatically. Rather, contemporary literature suggests that CT influence learning primarily by affecting the cognitive orientations, through which students interpret and apply knowledge in collaborative educational cultures. Consequently, IPSA turns into a plausible socio-cognitive aspect that explains how CT-induced learning may be associated with sustained AE.

In this regard, researchers refer to well-established theories, such as Social Constructivist Learning Theory (Vygotsky, 1978; Powell and Kalina, 2009), Problem-based Learning Theory (Hmelo-Silver, 2004; Savery, 2015), and Student Engagement Theory (Fredricks et al., 2004). Specifically, the Social Constructivist Learning Theory proposes that meaningful learning emerges through collaborative interaction and shared knowledge construction rather than through passive exposure to technological resources (Powell and Kalina, 2009). Likewise, Problem-based Learning Theory argues that authentic technology-supported learning environments promote meaningful learning, only when students actively analyze complex problems from multiple disciplines (Hmelo-Silver, 2004; Savery, 2015). They evaluate competing perspectives and also collaboratively construct solutions. Student Engagement Theory describes that university students become cognitively and emotionally engaged when learning activities are perceived as intellectually challenging and socially authentic (Fredricks et al., 2004; Kahu, 2013; Kahn, 2014). This way, existing theoretical perspectives indicate that IPSA provide an important cognitive bridge between CT-induced learning experiences and sustained AE.

Nevertheless, existing empirical research consistently supports the educational importance of interdisciplinary thinking within collaborative learning environments. Rather than improving AE through technology alone, CT create opportunities for learners to investigate authentic problems and exchange disciplinary perspectives. Recent literatures have demonstrated that technology-enhanced problem-based learning strengthens analytical reasoning, interdisciplinary collaboration, and higher-order thinking by encouraging students to integrate diverse sources of knowledge while solving complex real-world problems (Zhou et al., 2021; Kwon et al., 2021; Hava and Koyunlu Unlu, 2021; Longo et al., 2024; Kim and Kwon, 2024; Montag et al., 2024). Also, Sun and Liu (2026) reported that computational thinking positively predicted students' attitudes across different educational contexts. Thus, CT cultivate interdisciplinary cognitive orientations.

More importantly, university students possessing stronger IPSA are more likely to demonstrate sustained AE, because they actively connect knowledge across disciplines and participate more deeply in collaborative inquiry. Structured literature review have consistently determined that educational technologies would be positively associated with AE primarily when they promoted active collaboration and meaningful cognitive participation instead of passive technology use (Bond et al., 2020; Bedenlier et al., 2020). Recent reviews of digitally mediated learning environments indicate that interactivity, collaborative inquiry, immediate feedback, and interdisciplinary learning opportunities enhance behavioral participation and emotional involvement (Bozbiyik et al., 2025). Consequently, IPSA strengthen students' willingness to explore complex problems collaboratively, thereby transforming technological opportunities into meaningful educational participation.

From a psychological perspective, IPSA also encourage learners to perceive academic tasks as intellectually meaningful and personally relevant. Students, who habitually integrate multiple disciplinary perspectives, are more willing to question assumptions, negotiate alternative solutions, persist when confronted with complex problems. These behaviors stimulate collaborative knowledge construction and sustained participation throughout technology-supported learning activities, thereby strengthening all three dimensions of AE, as discussed in Fredricks et al. (2004). Accordingly, IPSA represent an important socio-cognitive mechanism, through which digital learning environments develop deeper AE. Technology-supported collaborative learning promotes greater self-regulated learning and collaborative knowledge construction, while also developing meta-cognitive skills–outcomes that collectively serve to strengthen student AE (Abou-Khalil et al., 2021; Alam, 2022; Kim, 2024; Yan et al., 2024; Nguyen et al., 2024b; Rui and Jin, 2026).

About this, Chaudhry et al. (2024) demonstrates that AE is an important psychological process linking supportive learning environments with positive educational outcomes. Viewed together, CT contributes to AE primarily by improving cognitive orientations and collaborative learning processes that facilitate university students to engage meaningfully with academic tasks. Although previous investigations have independently demonstrated positive associations between CT, IPSA, and AE, existing articles have seldom examined IPSA as the explanatory mechanism linking CT with AE within technology-supported collaborative learning environments. Accordingly, this study proposes a hypothesis as below:

*H*_3_: Interdisciplinary problem-solving attitudes statistically account for the positive association between computational tools and academic engagement among university students in technology-supported collaborative learning environments.

### 2.4 Cultural values, leadership skills, and unique contributions

Even while CT provide the technological infrastructure for collaborative learning, technology often fail to explain why some collaborative learning groups demonstrate higher levels of coordination and learning effectiveness than others. Contemporary literature increasingly recognizes that collaborative learning is simultaneously a technological and socio-cultural process, in which students interact and exchange knowledge while working with peers from diverse backgrounds. Consequently, successful technology-supported collaborative learning depends on the CV that shape students' shared responsibility and interpersonal relationships within collaborative learning cultures.

Regarding this, the Vygotsky's Sociocultural Theory (Vygotsky, 1978; Edwards and Usher, 2007), Bandura's Social Learning Theory (Bandura, 1977), and the principles of Culturally Responsive Leadership (Beatty and Guthrie, 2024; Maia et al., 2024) provided stronger theoretical foundation. Vygotsky argued that higher-order learning develops through socially mediated interaction, where cultural norms and shared practices influence how learners construct knowledge and solve problems collaboratively. Likewise, Bandura's Social Learning Theory proposes that collaborative behaviors, including communication, leadership, and cooperation, are acquired through observation, modeling, reciprocal interaction, and social reinforcement within culturally embedded learning environments (Bandura, 1977). Extending these perspectives, culturally responsive leadership emphasizes that recognizing learners' diverse cultural identities creates inclusive educational environments that strengthen collaboration, leadership development, mutual respect, and equitable participation among university students (Beatty and Guthrie, 2024; Maia et al., 2024). Collectively, these theoretical perspectives imply that CV influence university students' interpersonal interactions along with their collaborative competencies for meaningful learning.

Notably, empirical evidences increasingly support the educational importance of CV within collaborative higher education. Many existing studies demonstrate that cultural orientations influence communication styles, cooperation, interpersonal trust, conflict management, and collective decision-making, all of which are fundamental for successful collaborative learning (Hofstede, 2001; Deardorff, 2006). More recent articles indicate that culturally diverse learning environments promote richer collaborative experiences by encouraging learners to integrate multiple disciplinary perspectives, thereby strengthening IPSA (Jordens et al., 2025; Haregu et al., 2024). Moreover, educational leadership research consistently reports that culturally inclusive learning environments strengthen students' leadership identity and their willingness to participate actively within learning communities (Beatty and Guthrie, 2024; Maia et al., 2024; Abdallah, 2024). Therefore, CV act as an important socio-psychological foundation underlying collaborative learning within contemporary higher education.

This scenario motivates the present study to focus on LS and UC, because they represent two complementary behavioral dimensions of collaborative learning. Specifically, LS show learners' ability to coordinate collaborative activities and facilitate communication by resolving disagreements and collective decision-making toward shared academic goals. In contrast, UC reflect learners' willingness to enrich collaborative learning by contributing disciplinary expertise and creative perspectives. Whereas leadership primarily facilitates the organization and coordination of collaborative learning processes, UC determine the diversity and intellectual quality of the knowledge. Together, these complementary competencies explain not only how collaborative learning is coordinated but also how collective knowledge is created and sustained within technology-supported learning environments.

Accordingly, this study examines how CV are associated with these two competencies. First, this study examines the relationship between CV and LS, followed by the relationship between CV and UC within technology-supported collaborative learning environments, discussed as follows:

#### 2.4.1 Cultural values and leadership skills

Building upon the foregoing theoretical perspectives, LS represent one of the most important collaborative competencies through which CV become reflected in students' learning behaviors. Within technology-supported collaborative learning environments, leadership extends beyond formal authority. Instead, this represents a shared collaborative process involving the coordination of group activities and collective decision-making through conflict management and peer motivation. Aforementioned leadership behaviors guide students to organize collaborative inquiry effectively while maintaining inclusive participation and shared responsibility throughout interdisciplinary learning. Consequently, LS constitutes an important behavioral aspect for university students' cultural orientations in collaborative educational cultures (Wang et al., 2024).

In educational psychology literature, culturally responsive learning environments facilitate development of LS by encouraging the appreciation of diverse perspectives and coordinated collaborative activities (Jiang et al., 2022). Beatty and Guthrie (2024) argued that culturally relevant leadership education strengthened students' leadership identity through self-awareness and inclusive interpersonal relationships, whereas Maia et al. (2024) showed culturally relevant leadership boosting leadership efficacy in facilitating group learning in higher education, all while valuing the diverse perspectives of their peers. Also, Abdallah (2024) reported that cultural dimensions significantly influenced LS development within digitally mediated higher education. Thus, university students' socio-cultural orientations remain important determinants of leadership even within technology-supported learning environments.

Beyond educational leadership research, empirical studies in this area of research support the relationship between CV and leadership competencies. Tyminski and Owens (2024) identified communication, collaboration, interpersonal competence, ethical judgement, and shared decision-making as essential leadership competencies required in contemporary higher education. Alkhamees and Durugbo (2025) found that organizational culture, leadership, and knowledge management jointly promoted organizational learning within higher education institutions. They showed the complementary roles of CV and LS in creating effective collaborative learning environments. Collectively, LS develop from individual abilities and also from culturally embedded collaborative experiences among university students.

The educational significance of culturally grounded leadership has become increasingly evident as CT reshape collaborative learning. Culturally responsive leadership becomes particularly important because this promotes trust and equitable participation among culturally diverse learners. Recent studies demonstrate that collaborative leadership positively influences engagement. Balanced participation and effective coordination within technology-supported learning environments play a major role in this matter (Jordens et al., 2025; Chung et al., 2015). This way, CV are theoretically expected to support successful digital collaboration by strengthening LS that facilitate meaningful interaction among group members.

Moreover, the proposed relationship is further supported by Transformational Leadership Theory (Bass, 1985), which suggests that leadership develops through shared vision, interpersonal trust, and collaborative motivation rather than through formal authority alone. Within culturally diverse learning environments, students who internalize values of cooperation, mutual respect, and inclusiveness are more likely to demonstrate transformational leadership behaviors that facilitate communication, inspire peers, and coordinate collective learning activities. Accordingly, CV provide an important behavioral foundation for the development of LS in technology-supported collaborative learning (Jiang et al., 2016).

Taken together, the existing theoretical and empirical literature consistently note that CV influence students' LS by shaping cooperation and collaborative decision-making. Students, who value inclusiveness and collective responsibility, are thus expected to demonstrate stronger LS while coordinating interdisciplinary learning activities. As there are very few studies to independently examine culturally responsive leadership and organizational leadership in collaborative learning, there are ample scopes to explore the direct relationship between CV and LS of university students within technology-supported collaborative learning environments. This persuades to frame the following hypothesis:

*H*_4*a*_: Cultural values are positively associated with leadership skills among university students in technology-supported collaborative learning environments.

#### 2.4.2 Cultural values and unique contributions

Complementing the coordinative role of LS, successful collaborative learning depends on students' willingness to make meaningful intellectual contributions in collective learning. Whereas leadership primarily facilitates the coordination of collaborative processes, UC reflect students' ability to enrich collaborative learning through original ideas and disciplinary expertise. Within technology-supported collaborative learning environments, these contributions promote knowledge integration and interdisciplinary collaboration, thereby enhancing the quality of collective learning. Consequently, UC represent a complementary behavioral expression, through which CV become reflected in students' academic behaviors.

Educational psychology literature increasingly recognizes that collaborative learning is highly effective only when learners actively contribute their experiences rather than functioning as passive recipients of information. From the perspectives of sociocultural and cooperative learning theories, culturally diverse learning environments encourage students to exchange complementary perspectives and negotiate alternative viewpoints. Recent empirical evidences indicate that students' cultural orientations influence communication via trust, knowledge sharing, conflict management, and collaborative participation (Deardorff, 2006). Also, studies in digital cooperation demonstrate that students with stronger cooperative mindsets participate more actively. Also, they perceive greater educational benefits, because they contribute more consistently to collaborative learning processes (Jordens et al., 2025).

The growing integration of CT has increased the importance of students' UC (Haregu et al., 2024). Contemporary digital learning environments facilitate collaborative writing, shared knowledge construction, peer feedback, and interdisciplinary communication; however, these technological affordances generate pedagogical value only when students actively exchange knowledge and participate in collaborative inquiry. Consistent with this perspective, Nguyen et al. (2024a) argued that technology-supported collaborative learning enhances educational outcomes primarily through active learner participation, while Abou-Khalil et al. (2021) reported that carefully designed online learning environments strengthened AE by promoting collaborative interaction and shared responsibility.

Moreover, Khoa and Huynh (2024) demonstrated that knowledge-intensive teamwork benefits from continuous knowledge sharing and active participation, whereas intercultural education research indicates that students with stronger intercultural competence contribute more constructively to collaborative learning by appreciating diverse viewpoints and integrating multiple disciplinary perspectives (Jiang et al., 2015). Besides, culturally responsive educational environments encourage higher education students to transform their diverse experiences into valuable educational resources through reflective insights (Beatty and Guthrie, 2024; Maia et al., 2024).

This relationship is additionally explained by the Knowledge Creation Theory of Nonaka and Takeuchi (1995), which argues that organizational and educational knowledge evolves through continuous interaction, knowledge sharing, and integration of diverse individual perspectives. Within collaborative learning environments, culturally diverse students contribute unique experiences, disciplinary expertise, and creative insights that enrich collective knowledge construction. Consequently, CV encourage university students to make distinctive intellectual contributions that increase the quality of collaborative learning.

Taken together, university students, whose CV promote mutual respect-based cooperation and appreciation of diversity, are more likely to contribute original ideas and innovative solutions during collaborative learning. These contributions enrich collective knowledge construction while strengthening the effectiveness of technology-supported collaborative learning. As majority of previous studies have seldom investigated the direct relationship between CV and students' UC within technology-supported collaborative learning environments, this study examines how culturally grounded learning orientations encourage university students to become active contributors to collaborative knowledge construction. Thus, this study proposes the following hypothesis:

*H*_4*b*_: Cultural values are positively associated with unique contributions among university students in technology-supported collaborative learning environments.

Above deliberation indicates that CV are associated with two complementary collaborative competencies, namely LS and UC. Although both emerge from university students' culturally grounded collaborative orientations, they represent distinct yet interconnected dimensions of collaborative learning. LS primarily reflect the coordinative dimension of collaboration by enabling students to facilitate communication and guide collective decision-making. In contrast, UC represent the generative dimension of collaboration by encouraging them to enrich collective learning through disciplinary expertise and creative problem-solving. Accordingly, while LS determines how collaborative learning processes are coordinated, UC determine the quality and diversity of knowledge generated. All these prompt this study to examine whether these complementary collaborative competencies statistically account for the positive association between CV and AE.

### 2.5 Leadership skills and unique contributions as explanatory pathways

Building upon the preceding discussion, LS represent a behavioral tool, through which culturally grounded learning orientations are translated into sustained AE. While CV may affect university students' communication and shared responsibility during collaborative learning, these values are unlikely to influence AE directly. Rather, those students become academically engaged when culturally informed collaborative behaviors help them to coordinate teamwork effectively and create inclusive learning environments. Consequently, LS constitute an important socio-behavioral pathway linking CV with students' AE in technology-supported collaborative learning.

#### 2.5.1 Leadership skills as explanatory pathway

Building on the positive link between CV and LS, this relationship emerges as a key driver of students' learning experiences and AE. Unlike traditional hierarchical conceptions of leadership, leadership within collaborative higher education is increasingly understood as a shared and distributed process through which students organize group activities, coordinate responsibilities, encourage participation, and collectively solve interdisciplinary problems (Yang et al., 2020). Such collaborative leadership promotes mutual trust, effective communication, shared accountability, and collective decision-making, thereby creating learning environments that encourage active academic participation.

Empirical evidence increasingly supports the positive relationship between LS and AE. Beatty and Guthrie Beatty and Guthrie (2024) argued that culturally relevant leadership education enhances students' leadership identity, collaborative confidence, and participation by encouraging inclusive interpersonal relationships. Likewise, Maia et al. (2024) demonstrated that culturally responsive leadership learning strengthens students' leadership efficacy and collaborative engagement through recognition of diverse cultural experiences. Abdallah (2024) reported that leadership development within digitally mediated higher education is significantly influenced by students' socio-cultural characteristics, suggesting that leadership competencies remain fundamental even within technology-supported learning environments.

Beyond leadership development, recent investigations demonstrate that collaborative leadership directly promotes AE. Leadership behaviors characterized by communication, facilitation, and shared decision-making encourage greater behavioral participation, deeper cognitive processing, and stronger emotional commitment to collaborative learning tasks. For example, recent studies reported that transformational and participatory LS are significantly associated with students' AE within higher education (Wang et al., 2024; Zhang et al., 2021). Likewise, emotional leadership has been shown to strengthen engagement through supportive interpersonal relationships and positive academic emotions, thereby encouraging students to remain actively involved throughout collaborative learning activities. Recent conceptual work further suggests that LS and AE reinforce one another through continuous cycles of collaborative participation, reflective learning, and leadership development, indicating that leadership functions not only as an educational outcome but also as an important mechanism supporting sustained engagement.

The importance of leadership becomes particularly evident within technology-supported collaborative learning environments. CT facilitate communication and information sharing; however, successful collaboration depends largely on students' ability to coordinate distributed teamwork, negotiate responsibilities, resolve disagreements, and maintain productive interaction despite reduced face-to-face communication (Tang et al., 2026). Consequently, leadership transforms technological opportunities into meaningful collaborative experiences by encouraging inclusive participation and collective responsibility. Consistent with this perspective, Tyminski and Owens (2024) identified communication, collaboration, ethical judgement, interpersonal competence, and shared decision-making as essential leadership competencies for contemporary higher education. Likewise, Alkhamees and Durugbo (2025) demonstrated that organizational culture, leadership, and knowledge management jointly strengthen organizational learning, highlighting LS as a central mechanism for effective collaborative learning.

The proposed role of LS is consistent with Self-Determination Theory (Deci and Ryan, 1985), which posits that students become more actively engaged when learning environments satisfy their needs for autonomy, competence, and relatedness. Leadership behaviors encourage students to coordinate collaborative activities, support peers, and participate in shared decision-making, thereby strengthening these fundamental psychological needs. Consequently, LS provide an important motivational mechanism through which culturally grounded collaborative values become associated with higher levels of AE (Tu et al., 2020).

Collectively, the foregoing theoretical and empirical evidence suggests that CV foster leadership behaviors characterized by cooperation, inclusiveness, mutual respect, and shared responsibility, while leadership subsequently promotes behavioral participation, cognitive investment, and emotional involvement in collaborative learning activities. LS therefore provide the behavioral mechanism through which culturally grounded collaborative orientations become translated into meaningful AE. Although previous studies have independently demonstrated positive associations between CV and leadership development, along with that between LS and AE, there is relatively limited empirical research examining LS as an explanatory mechanism linking CV with AE among university students within technology-supported collaborative learning environments. This way, the present study proposes the following hypothesis:

*H*_5*a*_: Leadership skills statistically account for the positive association between cultural values and academic engagement among university students in technology-supported collaborative learning environments.

#### 2.5.2 Unique contributions as explanatory pathway

Complementing the role of LS, students' UC represent a second collaborative mechanism through which CV are associated with AE. While leadership primarily facilitates the coordination of collaborative learning activities, UC reflect students' active intellectual participation through the sharing of disciplinary knowledge, creative ideas, constructive feedback, and innovative problem-solving approaches. Consequently, collaborative learning becomes educationally meaningful not only when students organize group activities effectively but also when they actively enrich collective knowledge construction through their individual contributions. Within technology-supported collaborative learning environments, such contributions are expected to transform culturally grounded collaborative orientations into sustained AE with academic activities (Zhang et al., 2019).

Educational psychology increasingly recognizes that meaningful AE emerges when students participate as active contributors rather than passive recipients of knowledge. Contemporary collaborative learning models describe learning as a process of co-construction in which learners continuously exchange perspectives, evaluate alternative viewpoints, and generate shared understanding through dialogue and reflection (Jiang and Chen, 2018). Accordingly, students who contribute distinctive ideas and disciplinary expertise become more deeply involved in collaborative inquiry because active participation strengthens cognitive processing, psychological ownership of learning, and commitment to collective academic goals. This perspective is consistent with Cooperative Learning Theory, which emphasizes that positive interdependence and individual accountability jointly encourage learners to contribute meaningfully while supporting collective success (Su et al., 2025; Chung et al., 2011).

Empirical evidence consistently demonstrates that students' active contributions positively influence AE across diverse educational contexts. Recent investigations indicate that collaborative learning environments characterized by frequent knowledge sharing, peer interaction, and collaborative problem-solving promote higher levels of engagement among university students. For example, Khoa and Huynh (2024) demonstrated that knowledge-intensive teamwork in higher education is strengthened through continuous knowledge sharing, collaborative communication, and active exchange of expertise, thereby improving both collective learning and students' AE. Likewise, Nguyen et al. (2024a) argued that computational technologies enhance learning outcomes primarily when students actively contribute to collaborative inquiry rather than merely using digital platforms for information access. Similarly, Abou-Khalil et al. (2021) reported that online learning environments became substantially more engaging when instructional activities encouraged students to participate actively, exchange ideas, and assume shared responsibility for collaborative learning.

The importance of students' UC becomes even more evident within culturally diverse learning environments. Educational psychology literature suggests that cultural diversity enhances collaborative learning because students contribute complementary experiences, disciplinary knowledge, and alternative perspectives that stimulate critical thinking, creativity, and interdisciplinary problem-solving. Learners who appreciate cultural diversity are generally more willing to exchange ideas, respect alternative viewpoints, and integrate multiple perspectives during collaborative inquiry. Consequently, culturally inclusive learning environments encourage students not only to participate more actively but also to generate original contributions that enrich collective knowledge construction. Consistent with this perspective, intercultural education research has shown that students possessing stronger intercultural competence demonstrate greater collaborative participation, richer classroom interaction, and more constructive knowledge-sharing behaviors than those with lower levels of intercultural competence (Haregu et al., 2024). Likewise, culturally responsive educational environments have been found to strengthen students' confidence to express original ideas while promoting inclusive participation and collaborative innovation (Beatty and Guthrie, 2024; Maia et al., 2024).

Technology-supported collaborative learning further reinforces the importance of students' UC. CT help collaborative writing, real-time communication, shared document development, peer feedback, and interdisciplinary interaction; however, these technological affordances become educationally valuable only when learners actively contribute meaningful knowledge throughout collaborative learning processes. Recent research consistently indicates that technology alone has limited pedagogical value unless accompanied by active student participation, collaborative knowledge construction, and continuous intellectual exchange (Nguyen et al., 2024a; Abou-Khalil et al., 2021; Jordens et al., 2025). Accordingly, students' UC constitute an important socio-cognitive mechanism through which collaborative technologies foster meaningful engagement rather than superficial participation.

The explanatory role of UC is further supported by Knowledge Building (Scardamalia and Bereiter, 2006; Chen and Hong, 2016), which proposes that meaningful learning emerges through the continual improvement of shared ideas generated by individual learners. Students who actively contribute original perspectives and disciplinary expertise become more cognitively invested in collaborative inquiry because their ideas directly influence collective knowledge construction. Consequently, UC represent an important mechanism linking culturally grounded collaborative participation with sustained AE.

Collectively, the foregoing theoretical and empirical evidence suggests that students whose CV promote cooperation, inclusiveness, mutual respect, and appreciation of diversity are more likely to contribute original ideas, disciplinary expertise, constructive feedback, and innovative solutions during collaborative learning. These distinctive contributions encourage deeper cognitive investment, stronger behavioral participation, and greater emotional commitment to academic activities, thereby strengthening overall AE. UC therefore represent a theoretically grounded collaborative mechanism, through which culturally grounded learning orientations become translated into meaningful educational participation.

Although previous studies have independently examined collaborative learning, knowledge sharing, cooperative participation, and intercultural competence, relatively limited empirical research has investigated UC as the explanatory mechanism linking CV with AE within technology-supported collaborative learning environments. Existing articles have generally treated collaborative participation as a broad construct without explicitly considering students' distinctive intellectual contributions as an independent collaborative competency. Examining UC as an indirect association therefore extends existing educational psychology literature by integrating CV and AE within a unified theoretical framework. Notably, The proposed relationship is also consistent with Cooperative Learning Theory (Johnson et al., 2014).

Furthermore, investigating LS and UC simultaneously enables comparison of two complementary collaborative mechanisms through which CV are associated with AE, thereby providing a more comprehensive understanding of collaborative learning processes among university students. These competencies were selected because they represent the coordinative and generative dimensions of collaborative learning, respectively. Accordingly, the present study proposes the following hypothesis:

*H*_5*b*_: Unique contributions statistically account for the positive association between cultural values and academic engagement among university students in technology-supported collaborative learning environments.

### 2.6 Conceptual framework

Based on the foregoing theoretical and empirical discussions, the present study proposes an integrated conceptual framework explaining how technological and socio-cultural factors are jointly associated with university students' AE within technology-supported collaborative learning environments. The framework synthesizes perspectives from Social Constructivist Learning Theory (Vygotsky, 1978; Powell and Kalina, 2009), Problem-based Learning Theory (Hmelo-Silver, 2004; Savery, 2015), Student Engagement Theory (Fredricks et al., 2004), Vygotsky's Sociocultural Theory (Vygotsky, 1978; Edwards and Usher, 2007), Bandura's Social Learning Theory (Bandura, 1977), and Collaborative Learning Theory (Laal and Ghodsi, 2012; Johnson and Johnson, 2009) to explain the complementary roles of CT and CV in supporting collaborative learning. Notably, Social Constructivist Learning Theory and Sociocultural Theory provide the overarching theoretical lens, while the remaining theories explain specific mechanisms.

The first component of the framework focuses on the technological dimension of collaborative learning. Guided by Social Constructivist Learning Theory and Problem-based Learning Theory, CT are expected to facilitate collaborative inquiry, interdisciplinary knowledge integration, and active participation by providing technology-supported learning environments that encourage students to analyze authentic problems, exchange diverse perspectives, and construct knowledge collectively. Building upon these theoretical arguments, IPSA are proposed as an important socio-cognitive mechanism through which CT are associated with AE. Accordingly, CT are hypothesized to be associated with AE both directly and indirectly through students' IPSA.

The second component of the framework emphasizes the socio-cultural dimension of collaborative learning. Drawing upon Sociocultural Theory (Vygotsky, 1978; Edwards and Usher, 2007), Social Learning Theory (Bandura, 1977), and culturally responsive leadership, the framework proposes that students' CV shape collaborative behaviors by influencing communication, cooperation, shared responsibility, and appreciation of diverse perspectives. These culturally grounded learning orientations are expected to strengthen two complementary collaborative competencies. LS represent the coordinative dimension of collaborative learning by facilitating communication, group coordination, conflict management, and collective decision-making. In contrast, UC represent the generative dimension of collaborative learning by encouraging students to contribute original ideas, disciplinary expertise, constructive feedback, and innovative solutions that enrich collective knowledge construction. The framework therefore proposes that LS and UC statistically account for the positive association between CV and AE.

Collectively, the proposed conceptual framework integrates technological, cognitive, behavioral, and socio-cultural perspectives within a unified educational psychology model. Rather than examining CT or CV independently, the framework recognizes that successful collaborative learning emerges through the interaction of technological affordances, interdisciplinary cognitive orientations, culturally grounded collaborative competencies, and students' active engagement in learning. By simultaneously examining two theoretically distinct explanatory pathways, the framework provides an integrated theoretical explanation of how technological and socio-cultural factors are jointly associated with AE among university students.

**Figure 1:**
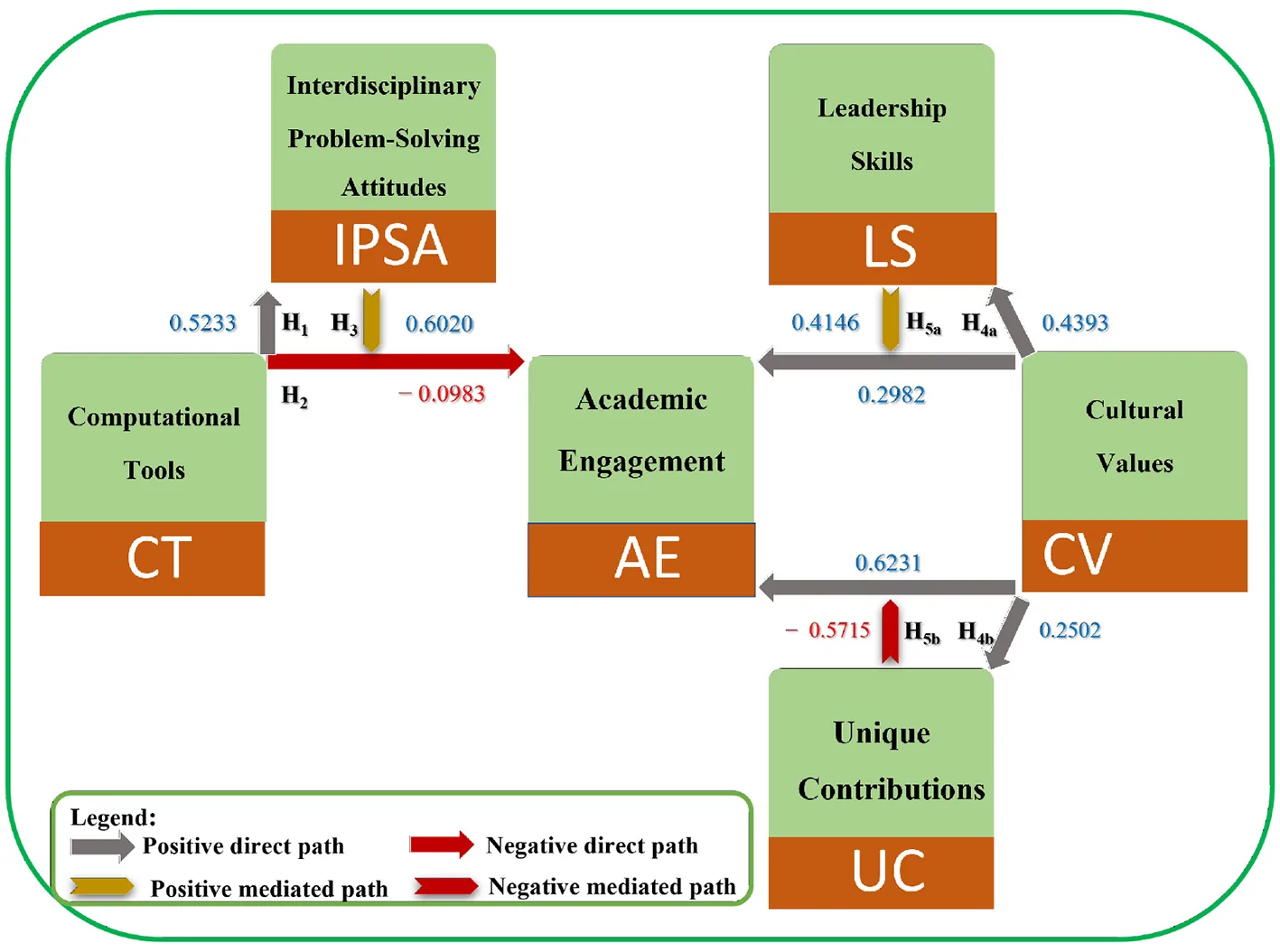
Graphical illustration of the proposed hypothesized relationships. Paths, i.e., CT → IPSA, CV → LS, CV → UC, IPSA → AE, LS → AE, and UC → AE, correspond to *B* in Table 9; horizontal predictor → AE paths, i.e., CT → AE and CV → AE, correspond to *DE* in Table 10.

Figure 1 illustrates the proposed conceptual framework together with the hypothesized relationships among the study constructs.

## 3 Methodology

This section outlines the participants of the primary data-based survey and provides a brief overview of the research instruments used, while also addressing data validation through control variables as follows:

### 3.1 Participants

The present study employed a questionnaire-based cross-sectional survey to collect primary data from university students that comprised students enrolled in public and private higher education institutions located within nine provinces of China, who had experience with technology-supported collaborative learning. Data collection was conducted between November 2024 and June 2025 to capture students' experiences with technology-supported collaborative learning. A total of 539 questionnaires were distributed. After excluding incomplete and inconsistent responses during data screening, 502 valid questionnaires were retained for analysis, yielding a usable questionnaire completion rate of 93.13%. The final sample included students with diverse demographic characteristics, including sex, ethnic background, career aspirations, and technology interests. Furthermore, participants were recruited using convenience sampling through voluntary participation via online survey links.

Data were collected using a self-administered online questionnaire hosted on a secure web-based survey platform. The survey link was disseminated through widely used Chinese online platforms, including Sojump, WeChat, and Wenjuanxing, which are commonly used communication channels among university students. Participants completed the questionnaire voluntarily and anonymously after receiving information regarding objectives and confidentiality of their responses. Clear instructions accompanied the questionnaire, and periodic reminders were distributed to encourage questionnaire completion among potential participants. Because participants were recruited through voluntary online participation using convenience sampling, the true response rate and non-response bias could not be estimated. Accordingly, the findings may be generalized with appropriate caution. Although the proposed framework is theoretically derived, cross-sectional designs do not establish temporal ordering or causal mediation, and therefore the estimated indirect effect (IE) should be interpreted cautiously (Maxwell and Cole, 2007).

#### 3.1.1 Justification of the cross-sectional research design

The present study employed a cross-sectional survey design to examine the theoretically derived associations among CT, CV, IPSA, LS, UC, and AE within technology-supported collaborative learning environments. A cross-sectional design was considered appropriate because the primary objective of this study was to examine relationships among theoretically specified psychological constructs within a large diverse sample of university students rather than to investigate developmental changes over time.

Consistent with educational psychology research, all constructs were measured during a single period of data collection using standardized self-report instruments. Although this design does not establish temporal ordering among current variables, this provides an appropriate framework for evaluating theoretically informed associations and statistically estimated indirect relationships among latent constructs. Accordingly, the proposed conceptual framework is grounded in established educational theories rather than temporal observations obtained from the data.

Furthermore, collecting responses from students enrolled in multiple public and private universities across nine provinces enhanced the diversity of current population and reduced the likelihood that the findings would be limited to a single institutional context. Nevertheless, consistent with recommendations in literature, current results may be interpreted as evidence supporting theoretically proposed indirect associations rather than temporal causal mechanisms. Future longitudinal, experimental, or cross-lagged investigations are therefore recommended to further evaluate the temporal ordering of the proposed relationships.

### 3.2 Ethical considerations

The present study involving human participants was reviewed and then approved by the Institutional Research Review Board of the Department of Chinese, Lyuliang University, Shanxi, China (IRRB Ref: CH/RB/2024/12; approval date: October 21, 2024). Prior to data collection, all participants provided informed, electronically written consent after receiving a clear explanation of current objectives, participation procedures, and their rights as research participants. This study was conducted in accordance with the ethical principles of the Declaration of Helsinki and complied with the applicable Chinese data protection regulations as well as the human research ethics policies of the participating institutions. Participation remained entirely voluntary, while anonymity and confidentiality of respondents were strictly maintained throughout this study. Participants were further informed that they could decline to answer any question or withdraw from the survey at any stage without penalty or the need to provide any justification. No personally identifiable information was collected, and all survey responses were analyzed in aggregated form solely for research purposes.

#### 3.2.1 Mitigation of common method bias

Given that the present study employed a cross-sectional, self-administered questionnaire in which all study variables were measured using self-reported responses from the same participants, the potential result of common method bias (CMB) was carefully addressed through both procedural and statistical approaches. Consistent with the recommendations of Podsakoff et al. (2003), several procedural remedies were incorporated during questionnaire design. Specifically, items representing different constructs were presented in separate sections and arranged in a randomized order, the wording of all items was kept clear and neutral to minimize response bias, and participants were assured of complete anonymity and confidentiality. Furthermore, respondents were informed that there were no correct or incorrect answers and were suggested to provide honest responses, thereby reducing evaluation apprehension and socially desirable responding.

Following data collection, multiple statistical procedures were undertaken to assess the potential role of CMB. Harman's single-factor test indicated that the first unrotated factor accounted for 50.38% of the total variance, indicating that some common method variance may be present (Wu and Wen, 2021). However, because contemporary methodological research has demonstrated that Harman's single-factor test alone is neither sufficiently sensitive nor conclusive for diagnosing CMB, additional diagnostic procedures were employed. Specifically, full collinearity variance inflation factors (VIF) and marker-variable analysis were conducted to provide complementary evidence. All VIF values remained below the recommended threshold of 3.3 (Kock and Lynn, 2012), while the inclusion of the marker variable did not produce any substantive changes in the estimated path coefficients. Collectively, the procedural safeguards together with the convergence of these statistical diagnostics indicate that, although CMB cannot be completely ruled out, this is unlikely to have materially affected the substantive relationships examined in the present study. Accordingly, the findings may be interpreted with appropriate caution, while recognizing that multiple complementary procedures were implemented to minimize the potential result of CMB. Although the first factor slightly exceeded the traditional heuristic threshold (Wu and Wen, 2021), recent methodological literature has demonstrated that Harman's single-factor test is insufficient as a stand-alone diagnostic.

### 3.3 Research instruments

All study constructs were measured using previously validated instruments that have demonstrated satisfactory psychometric properties in educational and psychological research. Since the original instruments were developed in English, the questionnaire was translated and culturally adapted for university students following a rigorous translation and back-translation procedure. Before the main survey, the translated questionnaire was pilot tested with 45 university students to evaluate its linguistic clarity, contextual appropriateness, response format, and completion time. Based on feedback from the pilot participants and expert reviewers, minor modifications were then made to the wording and sequencing of selected items without altering the conceptual meaning of the original constructs. The pilot participants were excluded from the final sample. Responses to all questionnaire items were recorded on a five-point Likert scale ranging from 1 (strongly disagree) to 5 (strongly agree), with higher scores indicating stronger agreement with the corresponding construct.

CT were measured using a six-item scale adapted from the Technology Acceptance Model (TAM) developed by Davis (1989). The instrument assessed students' perceptions regarding the usefulness, ease of use, and educational applicability of CT within collaborative learning environments. IPSA were assessed with scales adapted from Readiness for Interprofessional Learning Scale (RIPLS) (Parsell and Bligh, 1999) and Interdisciplinary Competence Scale (ICS) (Lattuca et al., 2013), thereby forming a 12-item instrument. The adapted items assessed university students' willingness to integrate knowledge across disciplinary boundaries, collaborate with peers from diverse academic backgrounds, and solve complex problems through interdisciplinary inquiry.

AE was assessed using the Student Engagement Instrument (SEI) proposed by Fredricks et al. (2004). This twenty-four-item instrument measured students' AE in academic activities and has been extensively applied in higher education research. CV were measured using Hofstede's Cultural Dimensions Scale (HCDS) (Hofstede, 2001). The twenty-item instrument evaluated key cultural dimensions, including individualism–collectivism, power distance, and uncertainty avoidance, thereby capturing students' socio-cultural orientations within collaborative learning environments. LS were assessed using the Multifactor Leadership Questionnaire (MLQ) developed by Bass (1990). The 45-item scale measured leadership behaviors associated with communication, motivation, coordination, and collaborative decision-making in group learning.

Since no single established instrument fully captured the intended construct of individual UC within collaborative learning environments, this study employed an author-adapted eight-item instrument. The instrument was developed by adapting conceptually relevant indicators from the Teamwork Quality (TWQ) framework (Hoegl and Gemuenden, 2001) and the Comprehensive Assessment of Team Member Effectiveness (CATME) framework (Loughry et al., 2007; Ohland et al., 2012). Item wording was modified to suit the context of collaborative learning among university students while preserving the conceptual meaning of the original indicators. The adapted instrument was reviewed for content relevance and clarity prior to administration. Also, the final instrument assessed students' original idea generation, knowledge sharing, constructive participation, collaborative problem-solving, and meaningful individual contributions within technology-supported collaborative learning environments, and demonstrated satisfactory psychometric properties in this study. The complete list of adapted UC items and their conceptual mapping to the source TWQ and CATME frameworks are provided in Appendix to enhance construct traceability.

The use of these established measurement instruments ensured comprehensive assessment of the proposed constructs while maintaining conceptual consistency with the theoretical framework developed in this study. Their combined application enabled the examination of the relationships among CT, IPSA, CV, LS, UC, and AE within technology-supported collaborative learning among university students. Notably, preliminary reliability analysis conducted on the pilot data indicated satisfactory internal consistency for all constructs (0.721 < Cronbach's α < 0.886), supporting the suitability of the questionnaire for the main survey.

### 3.4 Data validation and statistical analysis strategy

This study adopts a rigorous analytical strategy to ensure the validity and robustness of current empirical findings. Specifically, the present analyzes comprise data screening, assessment of distributional assumptions, reliability and validity evaluation, correlation analysis, robust regression modeling, and bootstrap-based indirect effect analysis. This multi-stage analytical procedure follows contemporary recommendations for behavioral and educational research (Peterson and Kim, 2013; Hayes, 2022). The analytical procedures are implemented sequentially so that assumptions associated with subsequent analyzes can be verified before hypothesis testing.

First, this study evaluates the internal consistency reliability by using Cronbach's α test (CAT). Notably, CAT is one of the most widely accepted indices of scale reliability in current research domain. Cronbach's α values greater than 0.70 indicate satisfactory internal consistency, whereas values exceeding 0.80 represent good reliability (Taber, 2018). Since Cronbach's α may underestimate construct reliability when indicator loadings differ, this study computes the composite reliability (CR). The CR is a more appropriate reliability estimate, and this incorporates standardized factor loadings (Peterson and Kim, 2013). CR values above 0.70 are usually indicative of satisfactory construct reliability.

Prior to hypothesis testing, this study examines the distributional characteristics of observed variables through skewness and kurtosis statistics. Following widely accepted recommendations, skewness values between −2 and +2 and kurtosis values between −7 and +7 indicate approximately normal distributions suitable for multivariate analyzes (Peterson and Kim, 2013; Kline, 2023). Because formal normality tests are highly sensitive to large sample sizes and may detect trivial departures from normality, distributional characteristics should be interpreted collectively rather than relying exclusively on significance tests. Any moderate deviations from normality are subsequently accommodated through robust statistical procedures during hypothesis testing.

Subsequently, this study evaluates the suitability of present research data for factor analysis. In this regard, the Kaiser–Meyer–Olkin (KMO) measure of sampling adequacy and Bartlett's sphericity test (BST) are well established measures. The KMO values assess whether the observed correlations among measurement items can support suitability of the data for construct validation. Also, BST evaluates whether the correlation matrix significantly differs from an identity matrix. Following established recommendations, KMO values exceeding 0.60 indicate adequate sampling adequacy, while a statistically significant BST (*p* < 0.05) may confirm that the observed variables share sufficient common variance (Kaiser, 1974; Peterson and Kim, 2013). These preliminary diagnostics are useful for supporting subsequent validation.

This research measures the construct validity by examining both convergent and discriminant validity. Convergent validity can be evaluated using the average variance extracted (AVE), which measures the proportion of variance captured by a latent construct relative to the variance attributable to measurement error. Following the recommendations of Fornell and Larcker (1981) and Peterson and Kim (2013), AVE values greater than 0.50 indicate that a construct explains more than half of the variance of its indicators, thereby providing evidence of satisfactory convergent validity. Notably, the internal consistency at the construct level are further examined using CR.

This study evaluates the discriminant validity with the heterotrait–monotrait ratio (HTMT) matrix, which is a more sensitive criterion than traditional approaches, such as the Fornell–Larcker criterion, for detecting insufficient discriminant validity among latent constructs. Following the recommendations of Henseler et al. (2015) and Franke and Sarstedt (2019), HTMT values below the conservative threshold of 0.85 indicate satisfactory discriminant validity, and confirm that the latent constructs are empirically distinguishable from one another.

Subsequently, this study explores the bivariate relationships among constructs through non-parametric correlation analysis. Because preliminary data screening indicates moderate departures from multivariate normality, this study computes both Spearman's rank correlation coefficient (ρ) and Kendall's rank correlation coefficient (τ). Spearman's ρ provides a robust measure of monotonic associations between variables, whereas Kendall's τ offers a more conservative estimate that is less sensitive to tied observations and small sampling fluctuations. Reporting both coefficients therefore provides a comprehensive assessment of the robustness of the observed bivariate relationships under non-parametric conditions (Kendall, 1948; Conover, 1999).

To ensure the robustness of the regression analyzes, multicollinearity was assessed through VIF statistics. VIF values below the conservative threshold of 3.3 indicate the absence of problematic full collinearity. Also, this implies that CMB is unlikely to substantially influence the estimated relationships (Kock, 2015). Notably, CMB need to be assessed, because all constructs were collected through a single self-reported questionnaire. In addition, influential observations are identified using Cook's distance, with observations exceeding the conventional threshold of 4/*n* receiving further examination (Cook, 1977; Nieuwenhuis et al., 2012). Evaluating influential observations ensures that the reported regression coefficients are not disproportionately driven by a small number of extreme cases, thereby enhancing the stability and robustness of the estimated relationships.

Beyond statistical significance testing, this study investigates the practical importance of estimated relationships by employing Cohen's effect size (*f*^2^), which quantifies the contribution of each predictor to the explained variance of the dependent variable. Reporting effect sizes complements statistical significance tests by indicating whether the observed relationships are of educational relevance rather than merely reflecting influence of sample size. Cohen (1988) stated that *f*^2^ values of 0.02, 0.15, and 0.35 would indicate small, medium, and large effect sizes, respectively.

To further evaluate the adequacy of the empirical analyzes, this study estimates the *post-hoc* statistical power. Statistical power represents the probability of correctly rejecting a false null hypothesis and therefore reflects the sensitivity of the study to detect the observed effects. Consistent with conventional recommendations, statistical power values exceeding 0.80 indicate that the analyzes possess adequate sensitivity to identify meaningful relationships while reducing the likelihood of Type II errors (Cohen, 1988; Faul et al., 2009). Accordingly, the combined reporting of statistical significance, effect sizes, and *post-hoc* statistical power provides a more comprehensive evaluation of both the statistical and practical robustness of the study findings.

Later, this study employs the ordinary least squares (OLS) regression together with bootstrap-based indirect effect analysis to evaluate the hypothesized relationships among constructs. Since the primary objective is to test the proposed hypotheses using observed composite scores, the robust OLS regression is regarded as an appropriate and parsimonious analytical approach. Moreover, preliminary data screening indicated moderate departures from normality, making robust estimation preferable to conventional OLS. Accordingly, heteroskedasticity-consistent (HC3) standard errors are adopted, because they provide more reliable coefficient estimates, standard errors (SE), and statistical inferences under conditions of heteroskedasticity and moderate violations of classical regression assumptions, particularly for behavioral and educational research (MacKinnon and White, 1985; Long and Ervin, 2000; Hayes, 2022). In this study, because preliminary diagnostics indicated moderate departures from normality, robust HC3 standard errors were used to obtain reliable statistical inference.

Accordingly, this study evaluates the direct hypotheses (i.e., H_1_, H_2_, H_4*a*_, and H_4*b*_) with the robust OLS regression with HC3 SE. The indirect hypotheses (i.e., H_3_, H_5*a*_, and H_5*b*_) are examined using non-parametric bootstrap estimation of indirect effects based on 5,000 bootstrap resamples. Bootstrap estimation is effective, because the sampling distribution of indirect effects is frequently non-normal. This makes resampling procedures more accurate and statistically powerful than traditional Sobel test (Preacher and Hayes, 2008; Hayes, 2022). Indirect effects are statistically significant, provided the corresponding 95% bootstrap confidence intervals (CI) excluded zero.

To facilitate interpretation beyond statistical significance, unstandardized regression coefficients (*B*), HC3 standard errors, *t*-statistics, *p*-values, CI, effect sizes (*f*^2^), and *post-hoc* statistical power are reported for the respective analyzes. Collectively, these complementary analytical procedures provide evidence regarding the reliability, validity, robustness, practical significance, and statistical sensitivity of the empirical findings, thereby ensuring that the reported hypothesis tests are methodologically rigorous and empirically defensible.

## 4 Results

This section reports the results of several statistical tests that were conducted on current research data, followed by an analysis of current findings as follows:

### 4.1 Demographic characteristics

Table 1 summarizes the demographic characteristics of the 502 university students who participated in this study. The sample comprised 258 males (51.39%) and 244 females (48.61%), indicating a nearly balanced distribution. Participants ranged in age from 19 to 27 years, with the largest proportion aged 21–23 years (41.24%, *n*:207), followed by those aged 24–27 years (37.85%, *n*:190) and 19–20 years (20.91%, *n*:105). Regarding career aspirations, 266 participants (52.98%) reported STEM-related career interests, whereas 236 (47.02%) indicated non-STEM career aspirations.

**Table 1:** Demographic characteristics of participants (*n*:502).

Category	*n* (%)
*Sex:*	
Female	244 (48.61%)
Male	258 (51.39%)
*Age (years):*	
19–20	105 (20.91%)
21–23	207 (41.24%)
24–27	190 (37.85%)
*Career aspiration:*	
STEM-related fields	266 (52.98%)
Non-STEM-related fields	236 (47.02%)
*Technology interest:*	
High interest	230 (45.82%)
Moderate interest	198 (39.44%)
Low interest	74 (14.74%)
*Ethnic background:*	
Han Chinese	366 (72.95%)
Ethnic minorities	136 (27.05%)
*Education level:*	
Undergraduate	374 (74.51%)
Postgraduate	128 (25.49%)

Furthermore, participants differed in their level of interest in technology, with 230 (45.82%) reporting high interest, 198 (39.44%) moderate interest, and 74 (14.74%) low interest. Current dataset included students from diverse ethnic backgrounds, comprising 366 Han Chinese students (72.95%) and 136 students (27.05%) from ethnic minority groups. In terms of educational level, 374 participants (74.51%) were undergraduate students and 128 (25.49%) were enrolled in postgraduate programs. Overall, the sample represented a demographically diverse group of university students from multiple higher education institutions.

### 4.2 Reliability and distributional assessment

Table 2 summarizes the psychometric and distributional characteristics of all study constructs. Cronbach's alpha coefficients ranged from 0.798 to 0.904, exceeding the recommended threshold of 0.70 (Taber, 2018), and thus indicating satisfactory to adequate internal consistency. Specifically, CT (α:0.865), IPSA (α:0.873), AE (α:0.871), CV (α:0.904), and LS (α:0.863) demonstrated adequate reliability, while UC also exhibited acceptable internal consistency (α:0.798). These findings indicate that all measurement instruments possessed adequate reliability for subsequent analyzes.

**Table 2:** Psychometric properties and distributional characteristics of current constructs (*n*:502).

Construct	Cronbach's α	Skewness	Kurtosis	KMO	Bartlett's χ^2^	*p*	AVE	CR
AE	0.871	–0.703	2.531	0.969	10167.975	0	0.618	0.975
UC	0.798	–1.417	4.929	0.647	2093.127	0	0.523	0.852
CT	0.865	–0.600	–0.916	0.939	3678.924	0	0.867	0.975
IPSA	0.873	–1.490	1.190	0.976	6845.814	0	0.786	0.978
CV	0.904	–1.350	2.635	0.914	2337.084	0	0.539	0.921
LS	0.863	–0.802	3.858	0.721	10574.882	0	0.504	0.968

The distributional characteristics of current variables were further examined using skewness and kurtosis statistics. Skewness values ranged from −0.600 to −1.490, indicating that participant responses were moderately concentrated toward the higher end of the measurement scales. Nevertheless, all skewness coefficients remained within the generally accepted range, thus suggesting no substantial departure from univariate normality.

Similarly, kurtosis values ranged from −0.916 to 4.929. Although some constructs exhibited relatively higher kurtosis values, all coefficients remained within acceptable limits for maximum likelihood estimation, indicating that the assumption of univariate normality was reasonably satisfied. This way, the reliability and distributional properties of current dataset supported its suitability for subsequent analyzes.

### 4.3 Assessment of sampling adequacy, convergent validity, and construct reliability

Table 2 provided the KMO measure of sampling adequacy together with BST χ^2^ values for all constructs. All constructs demonstrated satisfactory KMO values ranging from 0.647 to 0.976, while Bartlett's test of sphericity was statistically significant (*p* < 0.001) for every construct. These results indicated that the data were appropriate for factor analysis.

As AVE values ranged from 0.504 to 0.867, these exceeded the recommended threshold of 0.50 for all constructs (Henseler and Sarstedt, 2013), and thereby supporting convergent validity. CR coefficients ranged from 0.852 to 0.978, all exceeding the recommended threshold of 0.70 (Shrestha, 2021). Although LS exhibited the lowest KMO value (0.721), this exceeded the recommended threshold of 0.60 (Shrestha, 2021), while Bartlett's test remained highly significant (*p* < 0.001), which meant that the data were suitable for factor analysis.

Next, Table 3 summarized the explained variance and standardized factor loadings for all constructs. Explained variance ranged from 0.502 to 0.865 across current constructs, indicating that the latent variables accounted for a substantial proportion of the variance in their observed indicators. The factor loadings for CT ranged from 0.919 to 0.941, while IPSA demonstrated loadings between 0.835 and 0.913, indicating adequate indicator reliability. Although LS and UC exhibited comparatively lower minimum loadings, their overall loading patterns remained acceptable for construct measurement. The measurement indicators thereby demonstrated satisfactory convergent validity and construct reliability, as needed (Fornell and Larcker, 1981).

**Table 3:** Factor loading and explained variance analysis of constructs (*n*:502).

Item	Exp. var.	Min loading	Max loading	Mean loading
AE	0.617	0.685	0.861	0.785
UC	0.502	0.575	0.808	0.644
CT	0.865	0.919	0.941	0.931
IPSA	0.784	0.835	0.913	0.886
CV	0.589	0.668	0.809	0.734
LS	0.514	0.565	0.817	0.629

### 4.4 Discriminant validity analysis

Discriminant validity was evaluated using the HTMT matrix, and the results are presented in Table 4. Following the criterion recommended by Henseler et al. (2015), HTMT values below 0.90 indicate satisfactory discriminant validity between latent constructs. As shown in Table 4, all HTMT coefficients ranged from 0.3248 to 0.8194 and remained below the recommended threshold of 0.85 (Franke and Sarstedt, 2019), thereby supporting adequate discriminant validity for all study constructs.

**Table 4:** HTMT matrix for discriminant validity assessment (*n*:502).

Item	CT	IPSA	AE	CV	LS	UC
CT	1	0.8058	0.7616	0.5911	0.4710	0.3248
IPSA	0.8058	1	0.8091	0.7535	0.6239	0.4522
AE	0.7616	0.8091	1	0.8194	0.8108	0.6206
CV	0.5911	0.7535	0.8194	1	0.7802	0.7533
LS	0.4710	0.6239	0.8108	0.7802	1	0.8022
UC	0.3248	0.4522	0.6206	0.7533	0.8022	1

The highest HTMT values were observed between AE and CV (0.8194), AE and LS (0.8108), IPSA and AE (0.8091), and LS and UC (0.8022). Although these construct pairs exhibited comparatively stronger associations, all coefficients remained below the recommended threshold, indicating that the constructs retained adequate empirical distinctiveness. Conversely, relatively lower HTMT values were observed between CT and UC (0.3248), CT and LS (0.4710), and IPSA and UC (0.4522), providing further evidence of satisfactory discriminant validity across the proposed measurement framework.

Therefore, HTMT results demonstrate that the latent constructs are empirically distinguishable from one another. Taken together, the reliability, convergent validity, and discriminant validity assessments indicate that the measurement framework possesses satisfactory psychometric properties.

### 4.5 Correlation matrix

Because present variables did not fully satisfy the assumption of normality, Spearman's rank-order correlation and Kendall's tau correlation coefficients were computed to examine the bivariate associations among current constructs. Tables 5, 6 indicate positive associations among all study constructs, with comparable patterns observed across both correlation coefficients. Furthermore, all reported correlation coefficients were statistically significant (*p* < 0.001). Here, positive association was strongest between CT and IPSA (Spearman's ρ:0.811; Kendall's τ:0.804). Furthermore, IPSA exhibited an adequate positive association with AE (Spearman's ρ:0.867; Kendall's τ:0.806).

**Table 5:** Spearman's rank-order correlation matrix of current constructs (*n*:502).

Construct	CT	IPSA	AE	CV	LS	UC
CT	1.000	0.811	0.799	0.614	0.510	0.278
IPSA	0.811	1.000	0.867	0.672	0.560	0.303
AE	0.799	0.867	1.000	0.770	0.642	0.347
CV	0.614	0.672	0.770	1.000	0.799	0.444
LS	0.510	0.560	0.642	0.799	1.000	0.534
UC	0.278	0.303	0.347	0.444	0.534	1.000

**Table 6:** Kendall's τ correlation matrix of current constructs (*n*:502).

Construct	CT	IPSA	AE	CV	LS	UC
CT	1.000	0.804	0.691	0.518	0.432	0.234
IPSA	0.804	1.000	0.806	0.606	0.506	0.271
AE	0.691	0.806	1.000	0.723	0.603	0.324
CV	0.518	0.606	0.723	1.000	0.725	0.430
LS	0.432	0.506	0.603	0.725	1.000	0.522
UC	0.234	0.271	0.324	0.430	0.522	1.000

Likewise, AE was positively correlated with CV (Spearman's ρ:0.770; Kendall's τ:0.723) and LS (Spearman's ρ:0.642; Kendall's τ:0.603). CV exhibited adequate positive relationship with LS (Spearman: 0.799; Kendall: 0.725). In contrast, UC exhibited comparatively weaker, although consistently positive, correlations with the remaining study constructs. Thus, the correlation analyzes provided preliminary evidence of positive associations among current constructs.

### 4.6 Diagnostic assessment

Prior to evaluating CMB, the distributional properties of the data were examined using the Shapiro–Wilk and KS tests. Both the Shapiro–Wilk and KS tests were statistically significant for all constructs (*p* < 0.001), thereby indicating departures from strict normality. Accordingly, non-parametric correlation analyzes (i.e., Spearman's ρ and Kendall's τ) were employed to complement the subsequent analyzes. CMB was initially evaluated using Harman's single-factor test, in which the first unrotated factor accounted for 50.38% of the total variance.

Because Harman's single-factor test alone provides only a preliminary assessment of CMB, additional diagnostic procedures were performed. Following the recommendation of Kock (2015), full collinearity VIF were examined.

As shown in Table 7, all VIF values were below the recommended threshold of 3.3 (Kock and Lynn, 2012), ranging from 2.115 to 2.924. Taken together, the procedural remedies adopted during questionnaire design and the combined statistical evidence from Harman's single-factor test and the full collinearity VIF assessment suggest that, although some common method variance cannot be entirely excluded, this is unlikely to have materially affected the principal relationships examined in the present study. Accordingly, the hypothesized relationships were examined with appropriate caution, recognizing the inherent limitations of cross-sectional self-report data.

**Table 7:** Full VIF values of constructs (*n*:502).

Construct	VIF	Construct	VIF	Construct	VIF
CT	2.434	IPSA	2.827	UC	2.278
AE	2.924	CV	2.675	LS	2.115

In addition, Cook's distance was examined to identify potentially influential observations that could disproportionately affect the regression estimates. All Cook's distance values remained below the commonly recommended threshold of 4/*n* (Nieuwenhuis et al., 2012), indicating that no influential observations were detected in the dataset. Therefore, current results were unlikely to have been unduly affected by individual cases. Robust HC3 estimators were therefore employed subsequently.

### 4.7 Effect size and *post-hoc* analysis

To complement the hypothesis testing results, the practical significance of the estimated relationships was evaluated by computing Cohen's *f*^2^ effect sizes, while the adequacy of the sample size was assessed through *post-hoc* statistical power analysis. As presented in Table 8, the coefficient of determination (*R*^2^) ranged from 0.567 to 0.848 across the seven regression models, indicating that the proposed predictors explained a substantial proportion of variance in their respective endogenous constructs.

**Table 8:** Effect size and *post-hoc* statistical power of the hypothesized regression models (*n*:502).

Hypothesis	Relationship	*R* ^2^	Cohen's *f*^2^	Effect size	Power
H_1_	CT → IPSA	0.821	4.573	Large	>0.99
H_2_	CT → AE	0.580	1.382	Large	>0.99
H_3_	CT + IPSA → AE	0.848	5.574	Large	>0.99
H_4*a*_	CV → LS	0.775	3.439	Large	>0.99
H_4*b*_	CV → UC	0.567	1.312	Large	>0.99
H_5*a*_	CV + LS → AE	0.795	3.866	Large	>0.99
H_5*b*_	CV + UC → AE	0.797	3.918	Large	>0.99

Cohen's *f*^2^ values of 0.02, 0.15, and 0.35 indicate small, medium, and large effect sizes, respectively, following Cohen (1988). *Post-hoc* statistical power was estimated using the observed effect sizes, sample size (*n* = 502), and a significance level of α = 0.05 based on the non-central *F* distribution. Power values exceeding 0.80 are generally considered adequate, whereas all models in the present study achieved power greater than 0.99, indicating adequate sensitivity for detecting the observed effects.

The calculated Cohen's *f*^2^ values ranged from 1.312 to 5.574, which exceed the recommended benchmark for a large effect (*f*^2^ ≥ 0.35) proposed by Cohen (1988). In particular, the models, namely *H*_3_, *H*_5*a*_, and *H*_5*b*_, produced the largest effect sizes (*f*^2^:5.574, 3.866, and 3.918, respectively), indicating that the inclusion of this variable considerably enhanced the explanatory power of the corresponding regression models. Similarly, the direct-effect models (*H*_1_, *H*_2_, *H*_4*a*_, and *H*_4*b*_) also yielded large effect sizes, confirming the substantial contribution of CT and CV to their respective outcome variables.

To further evaluate the adequacy of current design, *post-hoc* statistical power was estimated using the observed effect sizes, the available sample size (*n* = 502), and a significance level of α = 0.05 based on the non-central *F* distribution. All regression models achieved statistical power greater than 0.99, indicating that the analyzes were sufficiently powered to detect the observed effects. These results suggest a negligible probability of committing a Type II error and provide additional evidence that the available sample size was more than adequate for testing the proposed hypotheses. Notably, although no formal priori power analysis was conducted before data collection, the final sample of 502 substantially exceeded commonly recommended minimum sample sizes for regression and bootstrap analysis of indirect associations in educational psychology research having multiple predictors.

Overall, the combination of very large effect sizes and consistently high statistical power provides further evidence for the robustness of the empirical findings. These results indicate that the observed relationships are not only statistically significant but also practically meaningful, thereby strengthening confidence in the stability and reliability of the estimated regression coefficients and the empirical support for the proposed theoretical framework.

### 4.8 Hypotheses validation: robust OLS regression and indirect effects estimation

Robust OLS regression with HC3 standard errors and bias-corrected bootstrap estimation of indirect associations were conducted to examine the hypothesized direct and indirect associations among variables. Prior to regression analysis, the assumptions of multicollinearity, influential observations, approximate normality, CMB, and heteroskedasticity were examined. Because moderate departures from normality were observed, heteroskedasticity-consistent HC3 SE together with bootstrap estimation were employed to obtain robust statistical inference. Accordingly, those results are provided in Tables 9, 10. Table 9 presents the robust OLS regression results used to evaluate the direct hypotheses H_1_, H_2_, H_4*a*_, and H_4*b*_, whereas Table 10 reports the bootstrap estimation of indirect effects used to evaluate the hypotheses H_3_, H_5*a*_, and H_5*b*_. Consistent with the cross-sectional design of this study, statistically significant indirect effects should be interpreted as indirect statistical associations rather than evidence of temporal ordering or causal mediation.

**Table 9:** Robust OLS regression results for the hypothesized regression models (*n*:502).

Relation	Variable	*B*	SE	*t*	*R* ^2^	Adj. *R*^2^	*F*-stat
CT → IPSA	Intercept	2.5955	0.044	58.708	0.821	0.820	2218.0
	CT	0.5233	0.011	47.095			
CT → AE	Intercept	4.0316	0.033	120.971	0.580	0.579	670.0
	CT	0.2168	0.008	25.885			
CT + IPSA → AE	IPSA	0.6020	0.033	18.017	0.848	0.847	359.7
CV → LS	Intercept	2.8080	0.053	52.782	0.775	0.774	1668.0
	CV	0.4393	0.011	40.841			
CV → UC	Intercept	3.7547	0.049	76.534	0.567	0.567	636.2
	CV	0.2502	0.010	25.223			
CV + LS → AE	LS	0.4146	0.406	1.022	0.795	0.794	133.0
CV + UC → AE	UC	−0.5715	0.401	−1.426	0.797	0.796	166.3

Unstandardized regression coefficients of direct associations CT → IPSA, CT → AE, CV → LS, and CV → UC have *p* < 0.001. SE, HC3 robust standard error.

**Table 10:** Bootstrapped estimates of the hypothesized indirect associations (*n*:502).

Relation	IE	DE	TE	95% bias-corrected bootstrap CI	*p*-value
*Indirect association via IPSA:*					
CT → IPSA → AE	0.3152	−0.0983	0.2169	[0.2833, 0.3452]	< 0.001
*Indirect association via LS:*					
CV → LS → AE	0.1808	0.2982	0.4790	[0.0605, 0.2948]	0.008
*Indirect association via UC:*					
CV → UC → AE	−0.1429	0.6231	0.4802	[−0.2150, −0.0671]	< 0.001

IE, indirect effect; DE, direct effect; TE, total effect; CI, confidence interval. Bias-corrected 95% bootstrap CI were estimated with 5,000 resamples.

Regarding hypothesis *H*_1_, this was examined using robust OLS regression with HC3 standard errors. Robust OLS regression analysis indicated that CT was positively associated with IPSA among university students. Specifically, CT exhibited an unstandardized regression coefficient (*B*) of 0.5233, with a robust standard error (SE) of 0.011 and a highly significant *t*-statistic of 47.095 (*p* < 0.001). The corresponding regression model explained 82.1% of the variance in IPSA (*R*^2^:0.821). Therefore, hypothesis *H*_1_ was supported.

Regarding hypothesis *H*_2_, robust OLS regression analysis revealed that CT had overall positive association with AE. Specifically, CT exhibited an unstandardized regression coefficient (*B*) of 0.2168, with a robust standard error (SE) of 0.008 and a statistically significant *t*-statistic of 25.885 (*p* < 0.001). The regression model accounted for 58.0% of the variance in AE (*R*^2^:0.580). Accordingly, hypothesis *H*_2_ was supported. Notably, subsequent bootstrap decomposition of this overall association (*H*_3_) revealed an inconsistent suppression pattern, whereby the positive overall association comprised a negative direct effect (DE) (DE: –0.0983) and a larger positive indirect association through IPSA (IE: 0.3152).

Next, hypothesis H_3_ was evaluated using bootstrap estimation of indirect effects based on 5,000 resamples. Bootstrapped indirect effects estimation revealed a statistically significant positive indirect association between CT and AE through IPSA (indirect effect, IE: 0.3152, *p* < 0.001), with the 95% bias-corrected bootstrap confidence interval excluding zero ([0.2833, 0.3452]). The corresponding DE of CT on AE was negative (DE: −0.0983), whereas the total effect (TE) remained positive (TE: 0.2169). IPSA was positively associated with AE (*B* = 0.6020, *p* < 0.001). The opposite directions of the indirect (positive) and direct (negative) effects are indicative of an inconsistent suppression pattern in the present cross-sectional dataset. Because all variables were measured simultaneously, these findings should be interpreted as statistically significant indirect associations rather than evidence of temporal ordering or causal mediation. These coefficients are also illustrated in Figure 1. Therefore, the results provide empirical support for the hypothesized indirect association between CT and AE via IPSA (Hypothesis *H*_3_).

Regarding hypothesis *H*_4*a*_, robust OLS regression analysis indicated that CV was positively associated with LS among university students. Specifically, CV exhibited an unstandardized regression coefficient (*B*) of 0.4393, with a robust standard error (SE) of 0.011 and a highly significant *t*-statistic of 40.841 (*p* < 0.001). The corresponding regression model explained 77.5% of the variance in LS (*R*^2^:0.775). Therefore, hypothesis *H*_4*a*_ was supported.

Regarding hypothesis *H*_4*b*_, robust OLS regression analysis revealed that CV was positively associated with UC among university students. Specifically, CV exhibited an unstandardized regression coefficient (*B*) of 0.2502, with a robust standard error (SE) of 0.010 and a statistically significant *t*-statistic of 25.223 (*p* < 0.001). The corresponding regression model explained 56.7% of the variance in UC (*R*^2^:0.567). Accordingly, hypothesis *H*_4*b*_ was supported.

Regarding hypothesis *H*_5*a*_, bootstrapped indirect effects estimation revealed a statistically significant positive indirect association between CV and AE through LS (indirect effect, IE: 0.1808, *p*:0.008), with the 95% bias-corrected bootstrap confidence interval excluding zero ([0.0605, 0.2948]). The corresponding DE of CV on AE remained positive (DE: 0.2982), while the TE was also positive (TE: 0.4790). Besides, LS were positively associated with AE (*B* = 0.4146), whereas UC showed a negative coefficient therein (*B* = −0.5715). These indicate a statistically significant indirect association between CV and AE through LS. However, because all study variables were measured simultaneously, the observed indirect association should not be interpreted as evidence of temporal ordering or causal mediation. Therefore, the results provide empirical support for the hypothesized indirect association between CV and AE via LS (Hypothesis *H*_5*a*_).

Regarding hypothesis *H*_5*b*_, bootstrapped indirect effects estimation revealed a statistically significant negative indirect association between CV and AE through UC (indirect effect, IE: −0.1429, *p* < 0.001), with the 95% bias-corrected bootstrap confidence interval excluding zero ([−0.2150, −0.0671]). The corresponding DE of CV on AE remained positive (DE: 0.6231), while the TE was also positive (TE: 0.4802). The opposite directions of the indirect (negative) and direct (positive) effects are indicative of a competitive (inconsistent) effects estimation pattern in the present cross-sectional dataset. Because all variables were measured simultaneously, these findings should be interpreted as statistically significant indirect associations. Although the indirect association was statistically significant, its direction was opposite to that hypothesized. Therefore, hypothesis *H*_5*b*_ was not supported as originally proposed.

#### 4.8.1 Summary

In short, the Table 11 summarizes the empirical evaluation of the proposed hypotheses together with the corresponding statistical procedures employed. The direct hypotheses, i.e., H_1_, H_2_, H_4*a*_, and H_4*b*_, were evaluated using robust OLS regression with HC3 SE, whereas the indirect hypotheses, i.e., H_3_, H_5*a*_, and H_5*b*_, were examined using non-parametric bootstrap estimation of IE based on 5,000 bootstrap resamples. The empirical findings supported six of the seven proposed hypotheses. Even though the proposed hypothesis H_5*b*_ was statistically significant, the indirect association was opposite to the hypothesized direction and therefore could not be supported. To facilitate interpretation, the following subsection discusses each hypothesis individually in relation to previous theoretical and empirical literature. Here, all the statistical analyzes were conducted using Python (version 3.13.8 Software Foundation, Wilmington, DE, USA) while employing the pandas, factor-analyzer, pingouin, statsmodels, semopy, scipy, and scikit-learn libraries.

**Table 11:** Summary of the validation of the proposed hypothesis (*n*:502).

Hypothesis	Relationship	Statistical analysis	Coefficient reported	*p*-value	Decision
H_1_	CT → IPSA	Robust OLS (HC3)	*B* = 0.5233	< 0.001	Supported
H_2_	CT → AE	Robust OLS (HC3)	*B* = 0.2168	< 0.001	Supported
H_3_	CT → IPSA → AE	Bootstrap (5000 resamples)	*IE* = 0.3152	< 0.001	Supported
H_4*a*_	CV → LS	Robust OLS (HC3)	*B* = 0.4393	< 0.001	Supported
H_4*b*_	CV → UC	Robust OLS (HC3)	*B* = 0.2502	< 0.001	Supported
H_5*a*_	CV → LS → AE	Bootstrap (5000 resamples)	*IE* = 0.1808	0.008	Supported
H_5*b*_	CV → UC → AE	Bootstrap (5000 resamples)	*IE* = −0.1429	< 0.001	Not supported^*a*^

^*a*^Although the IE was statistically significant, its direction was opposite to the hypothesized positive association; therefore, H_5*b*_ was not validated.

Direct hypotheses H_1_, H_2_, H_4*a*_, and H_4*b*_ are evaluated using robust OLS regression with HC3 SE and *B* values are displayed, whereas indirect hypotheses H_3_, H_5*a*_, and H_5*b*_ are examined using non-parametric bootstrap estimation of IE based on 5,000 bootstrap resamples and those IE values are displayed. Notably, Figure 1 shows *B* for direct predictor-indirect predictor paths and *DE* for paths leading to AE.

### 4.9 Comparison of present findings with literature

With respect to the validated hypotheses *H*_1_ to *H*_3_, CT were positively associated with IPSA and AE, while IPSA statistically accounted for the positive association between CT and AE. These findings support the core tenets of Social Constructivist Learning Theory (Vygotsky, 1978; Powell and Kalina, 2009), Problem-based Learning Theory (Hmelo-Silver, 2004; Savery, 2015), and Student Engagement Theory (Fredricks et al., 2004), which collectively posit that CT enhances learning outcomes through the facilitation of collaborative knowledge construction, underpinned by authentic problem-solving through active participation. CT transcend their function as mere instructional tools by creating interactive environments where university students can integrate cross-disciplinary knowledge and develop sustained AE within collaborative cultures.

The present findings are consistent with previous empirical studies. Like, Zhou et al. (2021), Kwon et al. (2021), and Hava and Koyunlu Unlu (2021) reported that technology-supported collaborative learning enhances interdisciplinary reasoning, analytical thinking, and collaborative problem-solving. Recent investigations involving explainable artificial intelligence and emerging educational technologies have likewise shown that CT strengthen higher-order cognitive skills and interdisciplinary learning orientations (Longo et al., 2024; Kim and Kwon, 2024; Montag et al., 2024). Furthermore, the positive relationship between CT and AE agrees with earlier findings that technology-supported learning environments improve students' engagement and learning motivation (Bond et al., 2020; Abou-Khalil et al., 2021; Alam, 2022). Also, the studies of (Nguyen et al., 2024b; Yan et al., 2024; Rui and Jin, 2026) determined analogous decisions. Therefore, current findings collectively support the pedagogically meaningful technology integration within higher education.

In addition, the significant indirect effect in hypothesis H_3_ extends existing educational psychology literature by identifying IPSA as an explanatory socio-cognitive mechanism linking CT with AE. This finding suggests that CT alone are insufficient to promote sustained AE. Rather, they become educationally meaningful when they cultivate interdisciplinary reasoning that enables students to analyze authentic problems collaboratively. While earlier studies have largely examined direct associations among CT, IPSA, and AE, relatively few have investigated how interdisciplinary cognitive orientations may translate technology-supported learning experiences into AE. Therefore, the present study contributes to literature by integrating technological, cognitive, and behavioral perspectives within a unified framework, as CT were found to develop deeper AE primarily by cultivating students' IPSA rather than through technological exposure alone.

Corresponding to the hypotheses *H*_4*a*_ and *H*_5*a*_, CV were found to be positively associated with LS, while LS statistically accounted for the positive association between CV and AE. These are consistent with Vygotsky's Sociocultural Theory (Vygotsky, 1978; Edwards and Usher, 2007) and Bandura's Social Learning Theory (Bandura, 1977), which posit that students' learning behaviors are shaped through socially affected interactions, cultural norms, and collaborative experiences. Within technology-supported collaborative learning environments, university students, who value cooperation and appreciate diverse perspectives, are more likely to develop leadership competencies. Culturally grounded collaborative norms strengthen students' willingness to coordinate group activities, facilitate communication, and assume responsibility during collaborative learning, thereby promoting effective leadership behaviors. The positive association between CV and LS corroborates existing studies discussing the importance of culturally responsive educational environments to develop inclusive leadership amid collaborative participation (Abdallah, 2024; Tyminski and Owens, 2024; Alkhamees and Durugbo, 2025).

Again, the significant indirect effect observed for H_5*a*_ extends previous educational psychology research by identifying LS as the behavioral mechanism through which CV contribute to AE. Whereas previous studies primarily treated LS as an educational outcome, this study demonstrates that LS also function as an explanatory behavioral mechanism linking CV with AE. This interpretation is consistent with recent articles emphasizing that collaborative leadership facilitates sustained participation and collective learning in higher education (Jordens et al., 2025; Zhang et al., 2024; Alkhamees and Durugbo, 2025). This way, this study extends existing literature through an integrated socio-cultural perspective of collaborative learning.

The hypothesis H_4*b*_ indicated CV positively associated with UC of university students within technology-supported collaborative learning environments. This finding is consistent with Sociocultural Theory (Edwards and Usher, 2007; Vygotsky, 1978) and Collaborative Learning Theory (Laal and Ghodsi, 2012; Johnson and Johnson, 2009), which suggest that culturally inclusive learning environments encourage students to contribute diverse knowledge and disciplinary expertise during collaborative inquiry. Students, who value cultural diversity and cooperative learning, are more willing to engage in constructive dialogue and enrich collective knowledge construction.

This way, results are in agreement with recent literature demonstrating that culturally responsive educational environments promote knowledge sharing and creative problem-solving (Haregu et al., 2024; Khoa and Huynh, 2024; Beatty and Guthrie, 2024; Maia et al., 2024). Likewise, intercultural education research discusses that students possessing stronger cultural awareness contribute more actively to collaborative learning because they value diverse viewpoints and regard their own experiences as valuable educational resources (Thinh, 2025). Therefore, this study reinforces the proposition that culturally grounded learning orientations encourage meaningful individual contributions within collaborative learning environments.

Nevertheless, contrary to expectations, the proposed hypothesis H_5*b*_ was not supported, as UC did not positively account for the relationship between CV and AE. Although university students with stronger CV were more likely to contribute original ideas and disciplinary knowledge, these individual contributions did not translate into higher AE after accounting for other variables in the proposed framework. This finding partially contrasts with previous research suggesting that knowledge sharing and active participation generally strengthen collaborative learning and AE (Johnson et al., 2014; Slavin, 2014; Khoa and Huynh, 2024).

One plausible explanation in this regard is that individual intellectual contributions alone may be insufficient to sustain engagement unless they are effectively coordinated through leadership, communication, and shared responsibility within collaborative groups. In the context of Chinese higher education, where collaborative learning often emphasizes collective coordination and group harmony, leadership behaviors may exert a stronger influence on AE than individual contributions. Consequently, the present findings indicate that LS constitute a stronger collaborative mechanism than UC in explaining how culturally grounded learning orientations are associated with students' AE within technology-supported collaborative learning environments.

Overall, the present findings demonstrate that technological affordances and socio-cultural learning processes complement one another in promoting AE within technology-supported collaborative learning environments. Whereas CT were primarily associated with engagement through IPSA, CV exert their influence largely through collaborative leadership. Collectively, these findings provide an integrated explanation of how cognitive and socio-cultural mechanisms jointly contribute to students' AE, thereby extending contemporary educational psychology literature on collaborative learning.

## 5 Discussion

This section derives implications along with discusses global implications of current insights. Also, this provides the limitations of present research and thus outlines directions for future research.

### 5.1 Theoretical implications

The present study makes several theoretical contributions to educational psychology and technology-supported collaborative learning literature. First, this integrates Social Constructivist Learning Theory (Vygotsky, 1978; Powell and Kalina, 2009), Problem-based Learning Theory (Hmelo-Silver, 2004; Savery, 2015), Student Engagement Theory (Fredricks et al., 2004), Vygotsky's Sociocultural Theory (Vygotsky, 1978; Edwards and Usher, 2007), Bandura's Social Learning Theory (Bandura, 1977), and Collaborative Learning Theory (Laal and Ghodsi, 2012; Johnson and Johnson, 2009) into a unified conceptual framework explaining university students' AE. Rather than examining technological and socio-cultural factors independently, the present framework demonstrates that CT and CV jointly contribute to AE through complementary cognitive and behavioral mechanisms.

Second, present findings extend existing literature by identifying IPSA and LS as two theoretically distinct explanatory pathways. While previous studies have generally examined the direct educational benefits of CT or culturally responsive learning environments, this study demonstrates that CT are associated with AE primarily through students' IPSA, whereas CV are associated with AE largely through LS. These findings provide a more comprehensive explanation of how technological affordances and socio-cultural learning orientations become translated into meaningful AE within collaborative learning environments.

Moreover, the unsupported hypothesis H_5*b*_ yields an additional theoretical insight. Although CV encouraged university students to make UC during collaborative learning, those contributions did not positively account for AE after considering the broader collaborative context. Thus, individual intellectual contributions alone may be insufficient to sustain engagement unless they are accompanied by effective coordination, communication, and shared leadership. Accordingly, this study highlights LS as a more influential collaborative mechanism than UC in explaining how culturally grounded learning orientations are associated with AE. Nevertheless, given the cross-sectional design, these indirect associations may be interpreted as theoretically informed statistical relationships rather than evidence of temporal or causal mediation.

### 5.2 Practical implications

Because the present study employed a cross-sectional design, the following implications may be interpreted as practical considerations derived from the observed statistical associations rather than as causal recommendations, mentioned as below:

• *University administrators:* Current findings suggest that university administrators may prioritize faculty development programs that facilitate the effective integration of CT into collaborative teaching and learning. The positive associations of CT on problem-solving and AE imply its transformative potential in classrooms. Faculty development programs in universities may focus on creating problem-solving tasks and leveraging digital platforms to promote collaborative learning. Particularly, well-trained instructors may optimize the benefits of CT tools, thereby enhancing student engagement and academic outcomes.

This study also promotes the importance of interdisciplinary collaboration in advancing problem-solving and engagement. Thus, universities may incentivize faculty and students to engage in interdisciplinary research and projects. This helps students develop diverse perspectives and stronger problem-solving skills, thereby better preparing them for the global job market. Universities may develop this type of environments by providing sufficient research funding and academic recognition to their faculties for interdisciplinary efforts.

Additionally, this study shows the association of CV on leadership behaviors in collaborative environments. Universities may consider implementing policies that cultivate an inclusive environment, while respecting and celebrating diverse cultural backgrounds. This study proposes that encouraging cultural sensitivity is associated with teamwork in diverse groups, thereby contributing to a more inclusive campus culture. Therefore, universities may consider supporting interdisciplinary teaching and institution-wide digital learning strategies that facilitate collaborative learning and technology-supported instruction.

• *Professors and educators:* This study proposes the creation of hybrid learning environments, where university students shall interact both face-to-face and virtually by using CT tools. This shows that CT is positively associated with university students' engagement, and hybrid learning may facilitate collaboration and participation. Hybrid learning environments provide flexibility, thus allowing university students to engage with material at their own pace while benefiting from face-to-face collaboration and instructor guidance. Chinese educators may adopt these practices as digitally supported learning continues to expand across higher education.

Additionally, since cross-cultural teamwork facilitates a greater appreciation for diversity and is associated with both leadership and collaboration skills, university professors may design collaborative learning activities that encourage interaction among students from different disciplinary and cultural backgrounds. In this regard, this study proposes to shape leadership behaviors by CV, and professors may generate cross-cultural teamwork in their classrooms. Professors in Chinese universities may replicate this by integrating group assignments that bring together students from different ethnicities and regions within China.

Given that LS are associated with AE, professors may incorporate leadership development activities into the curriculum. These may include group projects, peer mentoring, and student-led initiatives. Chinese universities may benefit by adopting these practices and thus better prepare their students for leadership roles in academic and professional settings.

• *Students:* Students may actively use CT tools to facilitate peer collaboration and improve academic performance. This study finds that CT is associated with problem-solving and engagement, and students may use tools, like collaborative online platforms, to work on group projects more effectively. Thus, Chinese students may better manage their group projects, improve communication, and streamline their workflow from using similar tools, thus leading to improved collaboration and problem-solving outcomes.

Current findings indicate that leadership behaviors, as shaped by CV, play an important role in AE. Students may adopt a growth mindset toward leadership, while embracing the opportunity to take leadership roles in collaborative tasks. Notably, a growth mindset toward leadership helps students recognize the value of leading by example and motivates them to contribute more actively to group work and academic tasks. Chinese students may implement similar practices by taking leadership roles in extracurricular activities or group assignments, which will enhance both personal and academic development.

University students may explore subjects outside their primary field of study. Since CT is positively associated with IPSA, they may benefit by developing knowledge in diverse fields. Universities may consider creating learning opportunities that encourage interdisciplinary collaboration among students. Expanding academic interests across disciplines may strengthen critical thinking, encourage creativity, and expose students to diverse approaches for solving complex problems.

• *Policymakers and governments:* Governments consider promoting digital education initiatives by integrating various CT tools across universities to enhance collaborative learning and student engagement. By ensuring access to modern technology and faculty training, the proposed approach helps students benefit from the potential of CT to improve AE and IPSA. Furthermore, supporting interdisciplinary education programs and research through grants and incentives foster collaboration across academic fields, thereby enhancing students' cognitive skills.

The government may also develop leadership training programs, while focusing on association of CV on leadership and ensuring students are equipped with essential skills for both academic and professional settings. Policies that emphasize cultural awareness and diversity in education, help students engage effectively in global teams. Besides, encouraging cross-border collaborations and framing university-industry partnerships may further bridge the gap between academia and the job market, which shall ensure that Chinese students acquire relevant skills. Lastly, strengthening mental health support programs will promote student wellbeing, further enhancing engagement and learning outcomes.

Although statistically significant indirect associations were observed, the present cross-sectional design does not permit conclusions regarding temporal ordering or causal mediation. Future longitudinal, experimental, or cross-lagged panel studies are required to verify the proposed theoretical pathways.

### 5.3 Global implications of current insights

Even though the primary data of this study had been collected in China, current insights hold relevance across geographies. By demonstrating positive statistical associations between CT and AE, this study suggests universities worldwide to consider prioritizing CT integration into their learning environments. Institutions in developing countries may adopt cost-effective digital tools and invest in faculty training to maximize educational outcomes.

Moreover, current findings on the role of CV in shaping leadership behaviors promote inclusive and culturally sensitive educational environments. Multicultural societies and international universities may implement policies that promote leadership training and cross-cultural competence, while helping students build teamwork and LS essential for globalized workplaces. Additionally, encouraging interdisciplinary education through funding cross-departmental programs and incentivizing collaborative research encourage higher education systems to strengthen CT-integration and conduct leadership training programs.

### 5.4 Limitations and future research directions

Like any empirical research, the present study has several limitations, some of which are as follows:

- The present study employed a cross-sectional research design in which all variables were measured simultaneously using self-reported responses. Consequently, temporal ordering among the constructs cannot be established, causal inferences cannot be drawn, and alternative theoretically plausible structural models (e.g., reversing variables) cannot be empirically distinguished (Maxwell and Cole, 2007; Maxwell et al., 2011). Therefore, the observed indirect effects should be interpreted as statistically estimated indirect associations rather than evidence of temporal or causal mediation.
  Future research should employ longitudinal, cross-lagged, or experimental designs to compare competing theoretical models and establish temporal precedence more rigorously.
- The present investigation was conducted within the cultural and educational context of Chinese higher education using a non-probability convenience sample. Consequently, the findings should be generalized cautiously and are primarily applicable to university students with characteristics similar to those represented in the present sample.
  Future studies should validate the proposed framework across different countries, educational systems, institutional settings, and more heterogeneous student populations to examine its cross-cultural robustness and external validity.
- Although several procedural and statistical remedies were implemented to minimize and assess common method bias, all constructs were measured using self-reported questionnaires administered to the same respondents. Accordingly, some degree of common method variance cannot be completely excluded.
  Future investigations may integrate multiple data sources, including instructor evaluations, peer assessments, learning analytics, and objective academic performance indicators, to further strengthen the validity of the findings.
- The present study conceptualized CT as a broad construct and did not differentiate among specific categories of CT, patterns of technology use, or levels of digital competence.
  Future research should investigate whether different computational technologies, technological infrastructure, digital literacy, and frequency of technology use exhibit differential associations with IPSA, LS, UC, and AE.
- Although the adapted UC instrument demonstrated satisfactory psychometric properties, this represents an author-adapted measure derived from the TWQ and CATME frameworks.
  Future studies should further examine its measurement invariance and external validity across different educational and cultural contexts.
- LS were identified as an important indirect pathway, even while this study did not distinguish among different leadership styles or collaborative leadership roles that may emerge during technology-supported collaborative learning.
  Future studies may examine transformational, transactional, shared, and distributed leadership styles to determine whether different leadership approaches differentially influence collaborative learning processes and AE.

Addressing these limitations will help establish the temporal robustness, external validity, and generalizability of the proposed conceptual framework.

## 6 Conclusions

The present study examined statistical associations among CT, IPSA, CV, LS, UC, and AE within technology-supported collaborative learning environments among university students in China. By integrating technological, cognitive, and socio-cultural perspectives within a single conceptual framework, this study contributed to educational psychology by providing a more comprehensive understanding of factors associated with university students' AE in digitally influenced higher education.

Overall, by merging technological and socio-cultural perspectives into a single analytical model, this study advanced the theoretical understanding of technology-supported collaborative learning. From a practical standpoint, these results underscored the need for higher education institutions to adopt targeted interventions that would reinforce collaborative skills and culturally inclusive teaching approaches.

## Data Availability

The raw data supporting the conclusions of this article will be made available by the authors, without undue reservation.

## A Appendix

### A.1 Questionnaire items

All questionnaire items were measured on a five-point Likert scale ranging from 1: *Strongly Disagree* to 5: *Strongly Agree*. Also, apart from IPSA and UC, other constructs were measured using their original validated instruments. Those were translated to Putonghua and culturally adapted following the standard translation, back-translation procedure.

#### A.1.1 Interdisciplinary problem-solving attitudes (IPSA)

IPSA was measured using the following 12 adapted items:

1. I enjoy solving academic problems by integrating knowledge from different disciplines.
2. I am willing to collaborate with students from different academic backgrounds to solve complex academic problems.
3. I believe that combining knowledge from different disciplines leads to better solutions.
4. I actively seek perspectives from other disciplines when completing collaborative learning tasks.
5. I value learning activities that require knowledge from multiple academic disciplines.
6. Collaborating with students from different academic backgrounds enhances my problem-solving ability.
7. I am receptive to viewpoints that differ from those of my own academic discipline.
8. Technology-supported collaborative learning encourages me to develop interdisciplinary solutions to academic problems.
9. I enjoy discussing academic challenges with students who possess different disciplinary expertise.
10. Integrating multiple disciplinary perspectives promotes more innovative solutions.
11. I feel confident participating in interdisciplinary teams to solve academic problems.
12. I prefer collaborative learning activities that involve interdisciplinary thinking.
  *Note:* IPSA items were adapted from established articles of (Parsell and Bligh 1999); (Lattuca et al. 2013).

**Unique contributions (UC)**

The UC was measured using the following adapted eight-item instrument:

1. I actively contribute useful ideas that help my group accomplish its learning tasks.
2. I willingly share my knowledge and expertise with other members of the group.
3. I make constructive suggestions that improve the quality of our group's work.
4. I contribute effectively to solving academic problems encountered during collaborative learning.
5. I complete my assigned responsibilities while also providing additional support when needed.
6. I encourage productive discussions by contributing relevant ideas and perspectives.
7. I make meaningful individual contributions that improve the overall performance of my group.
8. My contributions are recognized by my teammates as valuable to the successful completion of collaborative learning activities.

*Note:* The UC items were conceptually derived from TWQ dimensions, like Communication, Coordination, Mutual Support (Hoegl and Gemuenden, 2001) and CATME dimensions, like Contributing to the Team's Work, Interacting with Teammates, Expecting Quality, and Keeping the Team on Track (Loughry et al., 2007; Ohland et al., 2012). Because several adapted items integrate multiple conceptual indicators from both frameworks, the mapping presented below identifies the primary conceptual source for each item rather than implying an exclusive one-to-one correspondence (see, Table A1). The adaptation retained the conceptual meaning of the original TWQ and CATME dimensions while tailoring the wording to technology-supported collaborative learning among China's university students.

**Table A1:** Conceptual mapping of the adapted UC items.

Item	TWQ dimension	CATME dimension
UC_1_	Communication/coordination	Contributing to the team's work
UC_2_	Mutual support	Interacting with teammates
UC_3_	Communication	Expecting quality
UC_4_	Coordination	Keeping the team on track
UC_5_	Mutual support	Contributing to the team's work
UC_6_	Communication	Interacting with teammates
UC_7_	Coordination	Expecting quality
UC_8_	Mutual support	Keeping the team on track
